# HIV Infection Induces Extracellular Cathepsin B Uptake and Damage to Neurons

**DOI:** 10.1038/s41598-019-44463-1

**Published:** 2019-05-29

**Authors:** Yisel M. Cantres-Rosario, Sarah C. Ortiz-Rodríguez, Aemil G. Santos-Figueroa, Marines Plaud, Karla Negron, Bianca Cotto, Dianne Langford, Loyda M. Melendez

**Affiliations:** 1 University of Puerto Rico, Medical Sciences Campus, Department of Microbiology and Medical Zoology, San Juan, PR USA; 2 University of Puerto Rico, Bayamon Campus, Department of Biology, Bayamon, PR USA; 3 Lewis Katz School of Medicine at Temple University, Department of Neuroscience and Center for Neurovirology, Philadelphia, PA USA

**Keywords:** Mechanisms of disease, HIV infections, Monocytes and macrophages, Cell death in the nervous system, Neurodegeneration

## Abstract

HIV-associated neurocognitive disorders prevail in 20–50 percent of infected individuals. Macrophages transmigrate through the blood brain barrier during HIV-1 infection, triggering neuronal dysfunction. HIV-infected macrophages secrete cathepsin B (CATB), and serum amyloid p component (SAPC), inducing neuronal apoptosis by an unknown mechanism. *We hypothesized that HIV infection facilitates CATB/SAPC secretion from macrophages followed by neuronal internalization, promoting dysfunction*. SK-N-SH neuronal cells were exposed to active recombinant histidine-tagged cathepsin B (His-CATB). His-CATB entry was tracked by intracellular flow cytometry, and neuronal dysfunction was verified by western blot. Macrophage-derived extracellular vesicles (EVs) were tested for the presence of CATB and SAPC. Neurons internalized His-CATB, an effect that was partially decreased by pre-treatment with anti-CATB antibody. Pre-treatment with CATB and SAPC antibodies decreased cleavage of caspase-3 and restored synaptophysin in neurons. Neurons exposed to macrophage-conditioned media differentially internalized His-CATB, dependent on the HIV replication levels. Finally, CATB and SAPC were secreted in EVs. We report for the first time that CATB is secreted from macrophages both free and in EVs, and is internalized by neurons. Moreover, HIV-replication levels modulate the amount of CATB neuronal uptake, and neuronal dysfunction can be decreased with CATB antibodies. In conclusion, the CATB/SAPC complex represents a novel target against HIV-associated neurocognitive disorders.

## Introduction

HIV-1 targets CD4+ cells from the immune system, including T lymphocytes, monocyte/macrophages and microglia. HIV-infected macrophages can infiltrate through the blood brain barrier (BBB) to the central nervous system (CNS), where they secrete viral and cellular proteins that trigger neuronal dysfunction and death^1,2^. HIV-infected patients develop neurocognitive impairment due to neuronal dysfunction, which in the presence of antiretroviral therapy can range from asymptomatic to mild symptoms, or in rare cases to HIV-associated dementia (HAD). The mechanisms underlying the neurological dysfunction observed during HIV-1 infection are still unclear. Moreover, although the introduction of combined anti-retroviral therapy (cART) has resulted in controlled viral replication and has decreased the progression to HAD, mild cognitive impairment prevails in 20–50% of the HIV patients^3–5^.

Cathepsin B (CATB), a lysosomal cysteine protease, is secreted by HIV-infected monocyte-derived macrophages (MDM)^6^ and interacts with serum amyloid p component (SAPC) at the extracellular level^7^. Both proteins induce apoptosis of SK-N-SH neuroblastoma cells that is reduced when the MDM supernatants or macrophage-conditioned media (MCM) is pre-treated with anti-CATB or anti-SAPC antibodies^7^. These two proteins are increased in post-mortem brain samples from deep frontal white matter of patients diagnosed with HIV-associated neurocognitive disorders (HAND) compared to tissues from patients with normal cognition. Moreover, CATB and SAPC co-localized with amyloid beta (Aβ) peptides in brain tissues from patients with history of HAND and Alzheimer’s disease. Therefore, *we hypothesize that HIV infection of macrophages facilitates CATB/SAPC secretion and neuronal internalization, promoting dysfunction and neurocognitive impairment*.

CATB can be an effector protease at the intracellular level via the activation of the TNF pathway^8,9^, but it is not known if extracellular CATB can trigger death receptor-induced apoptosis or be internalized by neurons. It is known that macrophage-secreted viral proteins such as Tat, Nef, and gp120 can be found inside neurons and are capable of inducing neuronal apoptosis^10–14^. CATB is also related to endosomal changes^15,16^, thus the role of this protein in HIV-induced neuronal dysfunction needs to be studied.

We tested our hypothesis by tracking histidine-tagged cathepsin B (His-CATB) for internalization by neurons *in vitro*, using intracellular flow cytometry staining and western analyses. Internalization of His-CATB was associated with its neurotoxic potential as shown by increased cleaved caspase-3 and decreased synaptophysin expression in SK-N-SH neuronal cells. Finally, we observed that CATB and SAPC are secreted from MDM in EVs labeled with exosome markers, which could represent an alternate mechanism of internalization and neurotoxicity.

## Results

### Neurons internalize cathepsin B

**Figure 1:**
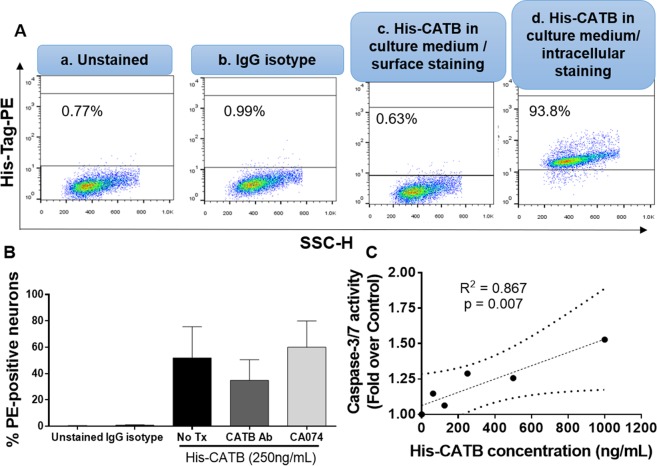
Neurons internalize His-CATB, which also triggers apoptosis in a concentration-dependent manner. Panel **A**: intracellular flow cytometry of SK-N-SH exposed to His-CATB at 250 ng/mL, based on previous CATB concentration measurements in uninfected and HIV-infected MCM by ELISA. Negative controls included no staining (a), staining with an IgG isotype (b), surface staining to ensure no cellular membrane attachment of the protein (c) and intracellular staining of one representative experiment (n = 4) (d). Panel **B**: Percentages of histidine tag-PE positive (PE+) neurons were graphed to compare the negative controls and treatments of His-CATB alone, or pre-treated with anti-cathepsin antibody (CATB Ab) or CA074 inhibitor. Panel **C**: Caspase-3/7 activity was measured by luminometry in neurons exposed to His-CATB in increasing concentrations. Caspase-3/7 activity is represented as percentage of control (untreated cells) in relative luminiscence units. Data presented as mean ± SEM.

CATB was internalized by neurons exposed to His-CATB in serum-free neuronal culture media (Fig. 1A; average of Phycoerythrin (PE) tag positive neurons = 52.0% from four independent experiments). Pre-treatment of media with anti-CATB antibody decreased the percentage of PE-positive neurons to 34.9%, while CA074 inhibitor had no effect in internalization (60.1%) (Fig. 1B). To determine the cellular localization of CATB in neurons after internalization, the percentage of PE-positive neurons was determined after setting the gates by first measuring fluorescence in treated cells unstained and stained with an IgG isotype control (Fig. 1A, panels a and b; quantification in Fig. 1B). Surface staining demonstrated that His-CATB is not at the neuronal membrane (Fig. 1A, panel c), but appears to be intracellular. Caspase-3/7 activity in neurons increased with higher concentrations of His-CATB (ranging from 0 to 1,000 ng/mL), with a positive correlation of R^2^ = 0.867 (p = 0.007) (Fig. 1C). To address His-CATB internalization, we measured the expression of mannose-6-phosphate receptor (M6PR) in untreated SK-N-SH neurons by flow cytometry. We chose this receptor based on previous literature from groups reporting that CATB uses M6PR to traffic within different cellular compartments^17–19^. Intracellular levels of M6PR (82.3%) were much higher than surface levels (8.1%) (Supplementary Fig. 1A) supporting data shown in Fig. 1A, panel c. Moreover, exposure to MCM for 24 hours increased the expression of M6PR in SK-N-SH over the untreated control (Supplementary Fig. 1B).

### Cathepsin B triggers neuronal dysfunction

**Figure 2:**
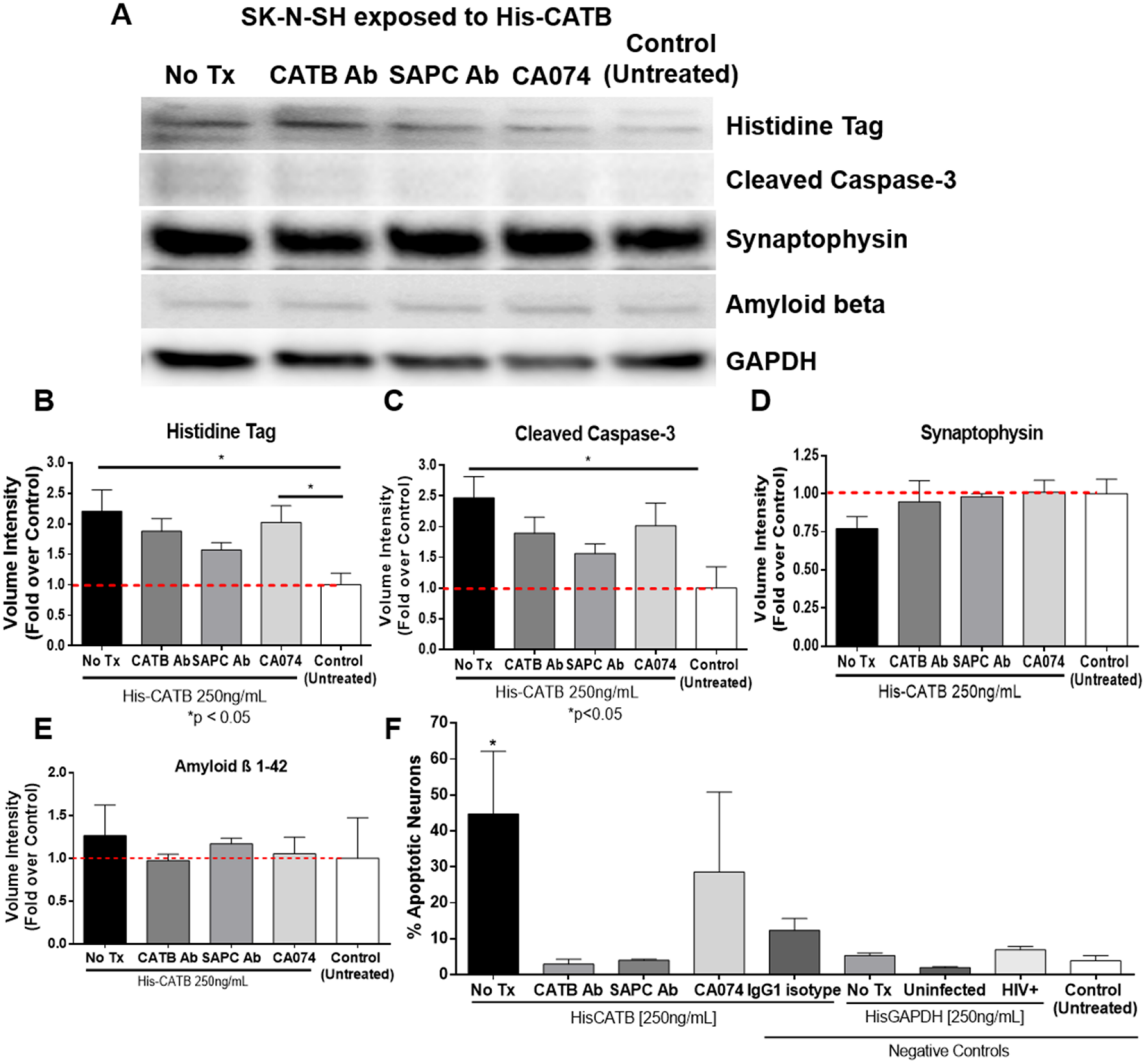
Effect of anti-CATB, SAPC antibodies, and CA074 on neuronal function upon exposure to His-CATB. SK-N-SH untreated or exposed to His-CATB 250 ng/mL alone (No Tx), or pre-treated with cathepsin B antibody (CATB Ab), SAPC antibody (SAPC Ab) and CA074, were lysed and compared by western blot (**A**). Densitometry analyses for volume intensity normalized against GAPDH of histidine tagged cathepsin B (**B**), cleaved caspase-3 (**C**), synaptophysin (**D**), and amyloid beta (**E**) expressed as fold over the control (untreated neurons) for comparison. For visualization purposes, the western blot pictures were cropped, but the original complete pictures were included in the supplementary information. Data presented as mean ± SEM of n = 3 experiments. SK-N-SH untreated or exposed to His-CATB 250 ng/mL alone (No Tx), or pre-treated with CATB antibody (CATB Ab), SAPC antibody (SAPC Ab), CA074 and IgG1 isotype control were fixed and stained with *in situ* cell death fluorescein TUNEL assay (**F**). At least three images were acquired for each condition. Green fluorescent nuclei were counted and divided by the total number of neurons (all DAPI-positive nuclei, blue) to express results as percentage of apoptotic neurons, using the ImageJ software (NIH).

Western analyses confirmed that neuronal cells internalized His-CATB *in vitro* (2.23-fold; p = 0.0156 compared to untreated cells) (Fig. 2A,B). Pre-treatment with CATB or SAPC antibodies decreased the volume of histidine tag to 1.88-fold and 1.56-fold respectively, while CA074 had no effect, as previously demonstrated by flow cytometry. Treatment of SK-N-SH with His-CATB triggered the cleavage of caspase-3 to 2.47-fold over the untreated cells (p = 0.0286 compared to untreated control cells). Cleavage of caspase-3 was decreased by the pre-treatment with anti-CATB (1.89-fold over untreated control cells) or anti-SAPC (1.56-fold over untreated control neurons) antibodies (Fig. 2C). CATB inhibitor CA074 had no effect on caspase-3 in treated SK-N-SH. His-CATB exposure slightly decreased synaptophysin, a synapse marker, to 0.78-fold compared to untreated cells. However, all the pre-treatments recovered synapses to 0.97-fold for CATB antibody, 0.99-fold for SAPC antibody and 1.01-fold for CA074, over the untreated cells (Fig. 2D). Intracellular Aβ load was not significantly affected by His-CATB (1.3-fold), nor anti-CATB treatments, compared to the control (Fig. 2E). Since caspase-3 can be temporarily activated in cells under stress, we measured DNA fragmentation as an indicator of apoptosis by TUNEL assay (Fig. 2F). Apoptosis levels were higher in neuronal cells exposed to His-CATB (p = 0.0175) compared to the untreated. Anti-CATB and anti-SAPC antibodies were neuroprotective, while CA074 elicited a 28% of toxicity, although not significantly higher than the controls. His-CATB pre-treated with IgG1 isotype decreased apoptosis from 45% to 12%, although not statistically significantly. Exposure to His-GAPDH in culture media did not trigger apoptosis in neuronal cells in either uninfected or HIV-infected MCM. Glyceraldehyde 3-phosphate dehydrogenase (GAPDH) is a glycolysis enzyme. We chose this protein because its expression remains stable, thus serving as western blot loading control. However, its exogenous addition to neurons might be stabilizing or even improving the glycolysis process in cultured neurons, contrary to the cellular stress/apoptosis we were expecting, as opposed to an expected histidine tag negative control. Since SK-N-SH is a neuroblastoma cell line that can be differentiated into neurons, confirmatory experiments were conducted in human primary neurons. The cleaved caspase-3, the pre-synaptic vesicles marker synaptophysin, the post-synaptic vesicles marker PSD95, amyloid beta, and brain-derived neurotrophic factor (BDNF) were measured in primary neurons. (Supplementary Fig. 2A). Human primary neurons were exposed to the same His-CATB concentration (250 ng/mL) and treatments including anti-CATB and SAPC antibodies, CA074 inhibitor used for SK-N-SH cells, and the treatment of His-CATB with IgG1 isotype control was included. Although the expression patterns of the proteins: histidine tag, cleaved caspase-3, synaptophysin were very similar, none of the treatments affected the primary neurons in a statistically significant manner. In addition, activation of caspase 3 was not detected with any of the treatments by western blot. Human primary neurons internalized 1.6-fold more His-CATB than the untreated control, and PSD95 and BDNF were markedly reduced to 0.57 and 0.42-fold, respectively.

### Neuronal uptake of cathepsin B is variable in macrophage-conditioned media from different donors

**Figure 3:**
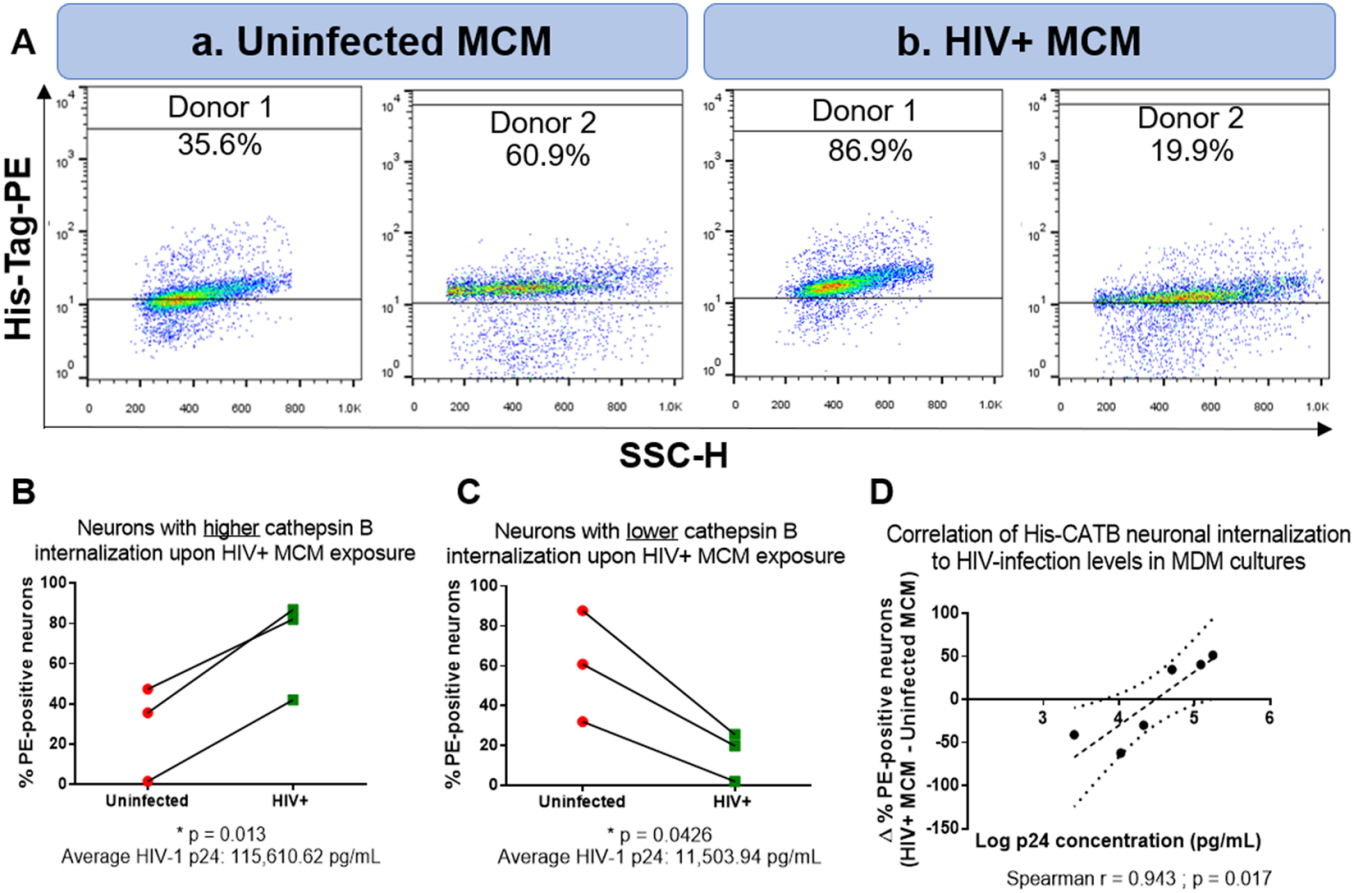
His-CATB internalization by neurons is dependent on the HIV-infectivity levels in macrophage-conditioned media. Panel **A**: SK-N-SH cells were exposed to His-CATB (250 ng/mL) diluted in uninfected MCM (a) or HIV-infected MCM (b). Data shown are representative of n = 6 MDM donors. Cells were detached and intracellular His-CATB was labeled with PE-conjugated anti-Histidine tag. The lower panels show donor-specific responses showing an increased percentage of PE-positive neurons upon exposure to HIV-infected MCM, n = 3 (**B**), while others had a lower percentage of PE-positive neurons upon exposure to HIV-infected MCM, compared to uninfected exposed to MCM, n = 3 (**C**). Spearman correlation of the change in PE-positive neurons (∆%PE+; HIV+ minus Uninfected) vs. the log of p24 concentration in the MDM supernatants at day 11 post-infection was performed as an indicator of HIV infectivity (**D**).

The percentage of His-CATB PE-positive neuronal cells was higher when treated with MCM from HIV infected MDM (70.33%; p = 0.013) with relatively high levels of HIV-1 p24 titer (115,610 pg/mL), compared to SK-N-SH treated with MCM from uninfected MDM (28.17%) (Fig. 3Aa,B). In contrast, when His-CATB was diluted in MCM from HIV-1 infection with relative low levels of HIV-1 p24 titer (11,503 pg/mL), the number of PE-positive neurons was lower (15.82%) than uninfected MCM (60.20%; p = 0.0426) (Fig. 3Ab,C). When combined, the two subpopulations of PE-positive neurons together are highly variable, and no trend could be determined (p = 0.958). A positive correlation was found between the positive change (∆)HIV-uninfected % PE-neurons with increasing levels of HIV-p24 (Spearman r = 0.943; p = 0.017; Fig. 3D) confirming the correlation between His-CATB internalization and the levels of HIV infection.

**Figure 4:**
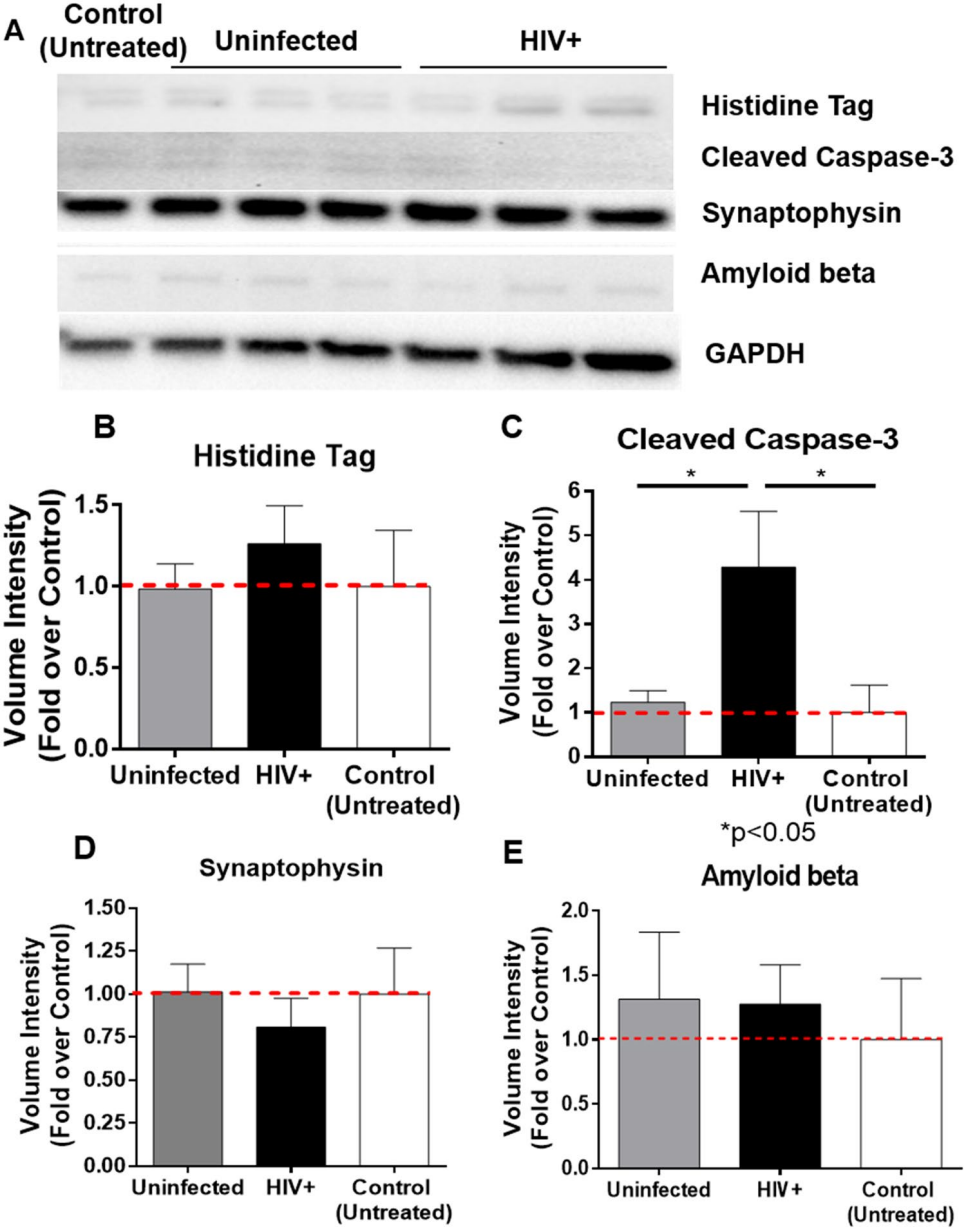
Neurons exposed to His-CATB in macrophage-conditioned media from day 12 post-infection. SK-N-SH untreated or exposed to His-CATB 250 ng/mL in uninfected or HIV-infected MCM from day 12 post-infection were lysed and analyzed for protein expression by western blot (**A**). Densitometry analyses for volume intensity normalized against GAPDH of histidine tagged CATB (**B**), cleaved caspase-3 (**C**), synaptophysin (**D**), and amyloid beta (**E**), expressed as fold over the control (untreated neurons) for comparison. For visualization purposes, the western blot pictures were cropped, but the original complete pictures were included in the supplementary information. Data presented as mean ± SEM of n = 2 experiments.

SK-N-SH neurons exposed to His-CATB in uninfected and HIV-infected MCM showed no statistical difference in the density of histidine tag by western analyses (Fig. 4A,B), confirming inter-donor variation in the internalization effect observed by flow cytometry. However, neuronal cells exposed to His-CATB in HIV-infected MCM tended to internalize more CATB (2.72-fold over the untreated control neurons) than uninfected MCM (1.38-fold). Interestingly, activation of caspase-3 is not affected in SK-N-SH exposed to His-CATB in uninfected MCM. However,. caspase-3 was activated by 4.3-fold in SK-N-SH exposed to His-CATB in HIV-infected MCM (p = 0.0241) compared to uninfected controls. Caspase-3 was also activated when compared to untreated neurons (p = 0.0268) (Fig. 4C). Synaptic vesicles were only slightly decreased when exposed to His-CATB in uninfected MCM (0.93-fold) and decreased to 0.75-fold in SK-N-SH exposed to His-CATB in HIV-infected MCM (Fig. 4D), with no statistical significance. Intracellular Aβ accumulation was not affected by His-CATB in uninfected or HIV-infected MCM, being only 1.3-fold over the untreated control (Fig. 4E). Altogether, CATB internalization and neuronal damage increased with His-CATB in HIV-infected MCM compared to serum-free neuronal culture media or in uninfected MCM. Experiments performed in primary rat cortical neurons also showed that His-CATB internalization, activation of caspase-3, and loss of synapses occurs in primary neurons exposed to HIV-infected MCM *in vitro* (Supplementary Fig. 2B). Similar to the results with human primary neurons, lack of statistically significant changes suggest that rat primary neurons are not affected to the same level as the neuroblastoma cells. However, rat primary cortical neurons exposed to HIV+ MCM exhibited increased cleaved caspase-3 (Supplementary Fig. 3A), and increased apoptosis (Supplementary Fig. 3B) that were reduced by anti-CATB and anti-SAPC antibodies. To elucidate if Aβ aggregation is a common mechanism of CATB and SAPC induction of neuronal dysfunction in HIV, we measured the levels of Aβ_1–42_ in rat primary cortical neurons exposed to HIV-infected MCM with or without anti-CATB, anti-SAPC antibodies, and CA074 inhibitor. Inhibition of CATB and SAPC reduced intracellular accumulation of Aβ peptides in rat cortical neurons exposed to HIV-infected MCM, representing a third mechanism by which CATB and SAPC might contribute to neuronal dysfunction upon HIV-1 infection of the CNS (Supplementary Fig. 3A). When compared with the SK-N-SH cell line exposed to uninfected and HIV-infected MCM without His-CATB, caspase-3 was found equally activated by uninfected and HIV-infected MCM, to the same levels of His-CATB in HIV-infected MCM. Interestingly, Aβ was significantly higher in SK-N-SH exposed to HIV-infected MCM than with His-CATB treatments and the untreated control (p < 0.005) (Supplementary Fig. 3C).

### Cathepsin B and SAPC are secreted from MDM in extracellular vesicles

**Figure 5:**
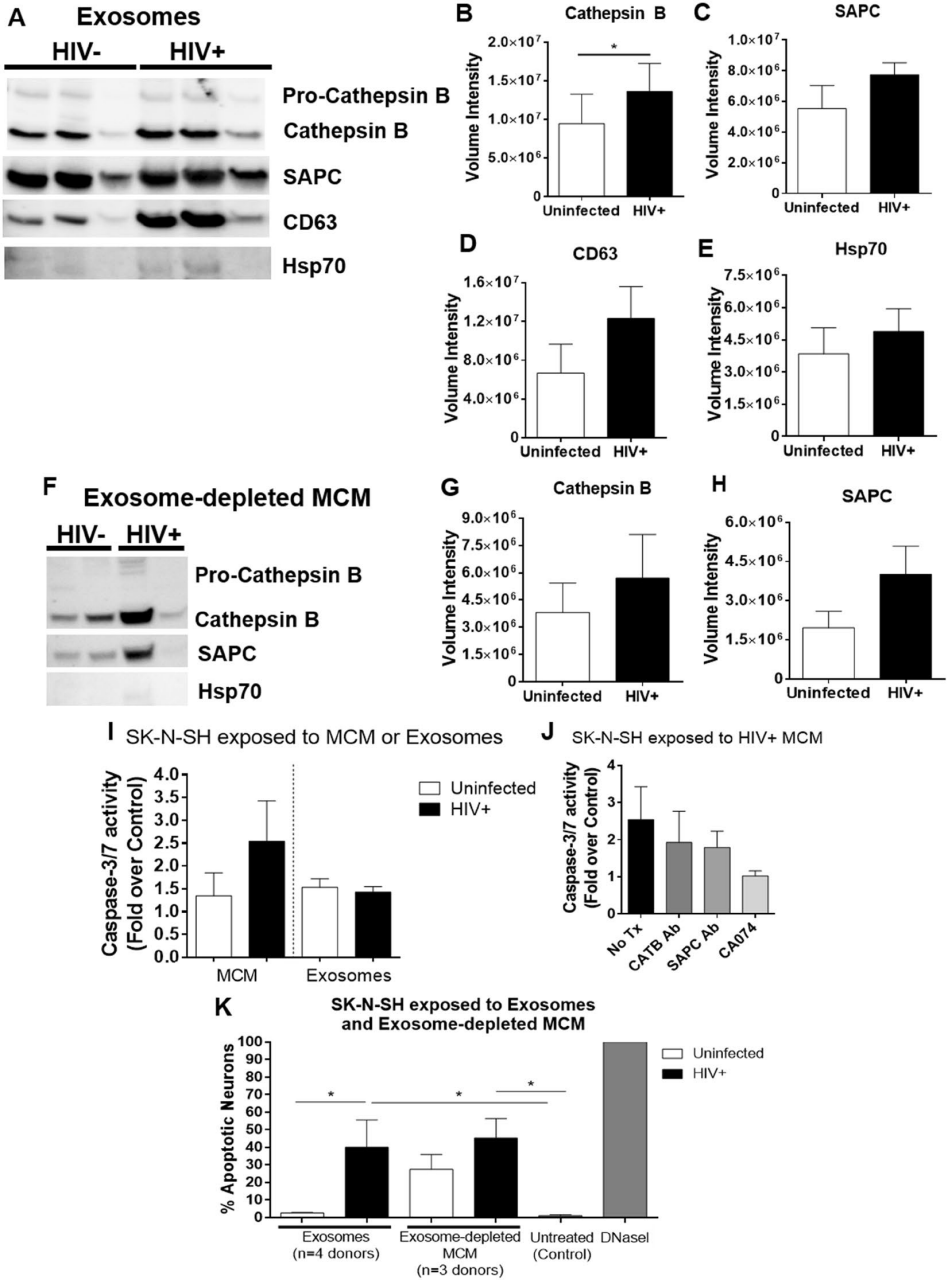
Cathepsin B/SAPC complex is secreted in macrophage-derived EVs. Total exosomes isolated from MCM at day 12 post-infection were analyzed by Western blot (**A**). Densitometry analyses of volume intensity for CATB (**B**), SAPC (**C**), CD63 (**D**) and Hsp70 (**E**) in EVs (n = 3). Western blot (**F**) and densitometry analyses of volume intensity for CATB (**G**) and SAPC (**H**) in exosome-depleted fractions (n = 4), where Hsp70 was not detected. For visualization purposes, the western blot pictures were cropped, but the original complete pictures were included in the supplementary information. Caspase-3/7 luminometric measurement in neurons exposed to MCM (n = 5) and isolated exosomes (n = 4) derived from uninfected and HIV-infected MDM from day 12 post-infection, for 24 hours (**I**). Caspase-3/7 measurement in SK-N-SH exposed to MCM (n = 4) from day 12 post-infection by luminescence was pre-treated with CATB antibodies (CATB Ab), SAPC antibodies (SAPC Ab) and CA074 (**J**). Data presented as mean ± SEM, or percentage of control (untreated SK-N-SH). SK-N-SH exposed to exosome and exosome-depleted fractions were fixed and stained with *in situ* cell death fluorescein TUNEL assay (**K**). At least three images were acquired for each condition. Green fluorescent nuclei were counted and divided by the total number of neurons (all DAPI-positive nuclei, blue) to express results as percentage of apoptotic neurons, using the ImageJ software (NIH).

EVs were isolated from MCM using a total exosome isolation reagent. Exosome-depleted and exosome-containing fractions were separated by centrifugation. The EV fractions isolated from MCM from four donors were characterized by nanoparticle tracking analysis (NTA). Finite track length analysis revealed that the mean size of the EVs from the four donors was 156.1 nm, with a mode of 83.3 nm (Supplementary Fig. 5). This value is similar to the size of exosomes, which is the portion of EVs smaller than 100 nm. Western analyses were used to measure CATB and SAPC, along with the exosome markers Hsp70 and CD63 (Fig. 5A,F). Pro-enzyme and mature forms of cathepsin B and SAPC are contained in exosomes. We observed increased CATB in EVs from HIV-infected MCM (p = 0.0177; Fig. 5B), with no differences in the exosome-depleted fraction (Fig. 5G). There were no differences in the secreted levels of SAPC (Fig. 5C,H) in either of the fractions. Caspase-3/7 activity was increased in EVs by 1.53-fold (uninfected) and 1.43-fold (HIV+) over the untreated neurons, with no statistical differences between uninfected and HIV-infected EVs (p = 0.88; Fig. 5I). The activity of caspase-3/7 triggered by EVs was similar to that triggered by uninfected MCM (1.34-fold over untreated neurons). HIV-infected MCM triggered a larger increase in caspase-3/7 activity (2.54-fold) over the untreated neurons or the exosomal fraction alone, indicating the extracellular proteins outside the EVs have higher pro-apoptotic potential upon HIV infection than EVs from uninfected MCM. Apoptosis triggered by HIV-infected MCM (2.54-fold over untreated neurons) was slightly decreased by CATB antibody (1.93-fold), SAPC antibody (1.79-fold) and CA074 (1.02-fold) (Fig. 5J). Following these three pre-treatments, we found that CA074 conferred the highest levels of protection when added to HIV-infected MCM. To further confirm that the activation of caspase-3 leads to apoptosis and is not a transient event, SK-N-SH were exposed to exosome and exosome-depleted fractions from uninfected and HIV-infected MCM (Fig. 5K). Exosomes from HIV-infected MCM triggered 40% apoptosis, compared to 2.6% exosomes from uninfected MCM (p = 0.0242) and 1% untreated control (p = 0.0415). The exosome-depleted fraction from HIV-infected MCM triggered 45% apoptosis (p = 0.0462 compared to untreated), and 27% uninfected MCM (Fig. 5K).

To assess the expression levels of SAPC and CATB proteins in the brains of HIV and AD patients, immunolabeling and quantification was conducted on frontal cortex of post-mortem tissue from the National Tissue Consortium. Increased CATB expression was observed in post-mortem brain from HIVE/HAD and AD patients compared to HIV patients with normal cognition (NC), asymptomatic neurocognitive impairment (ANI) or healthy controls (Supplementary Fig. 4, left panel). We also quantified levels of SAPC in control, NC, ANI, HIVE/HAD and AD but were not significantly different (Supplementary Fig. 4, middle panel). Interestingly, CATB co-localized with Aβ_1–42_ peptides in brain tissue from both AD and HIV-related neurodegenerative diseases^7^. Thus, we also quantified the expression of Aβ_1–42_ in the brains from these cases and observed significantly increased levels of Aβ_1–42_ NC, ANI, HIVE/HAD and AD compared to control tissues (Supplementary Fig. 4, right panel).

## Discussion

During HIV infection, neurons are susceptible to neurotoxic factors secreted by infected cells in the brain, including Tat and gp120 viral proteins, that have been found inside neurons^20,21^. We report for the first time the neuronal uptake of active recombinant CATB, a macrophage-derived neurotoxic enzyme. The process of internalization of CATB by neurons is not exclusive to MCM, because the recombinant protein was also internalized by neurons when exposed in serum-free neuronal culture media. We have demonstrated that MDM-secreted CATB upon HIV infection, interacts with SAPC and triggers neuronal apoptosis^7^. To determine if receptor-induced cell death triggers apoptosis, we tested the activation of caspase-3, a common effector for most apoptotic pathways, upon neuronal exposure to His-CATB. We confirmed that CATB is pro-apoptotic in a concentration-dependent manner, activating signaling pathways involving caspase-3. Moreover, internalization and apoptosis are decreased by CATB and SAPC antibodies, but not by the CATB inhibitor. These results led us to hypothesize that the mechanism of neuronal dysfunction triggered by CATB/SAPC complex does not rely on the active site of CATB, but in its interactions at the extracellular level and with the neurons. Human primary neurons show higher levels of apoptosis upon exposure to HIV+ microglia-conditioned media, which decreases after pre-treatment with anti-CATB antibody or with CATB inhibitor CA-074^22^. The addition of His-CATB in normal culture media did not trigger as much dysfunction in human primary neurons as observed in SK-N-SH. These results suggest that concentration of CATB and protein interactions in HIV+ macrophage or microglia conditioned-media are different and differentially affect neuronal function.

We demonstrated that the process of neuronal uptake of CATB depends on HIV replication levels. The high variability in the percentage of neurons internalizing CATB diluted in MCM may also depend on the variability in immune activation, genotype, and susceptibility to HIV infection observed among the MDM donors. Interestingly, we observed differences in neuronal behavior when neurons were exposed to His-CATB in neuronal culture media compared to MCM. His-CATB in neuronal media was 2.23-fold internalized, increased cleaved caspase-3 to 2.25-fold over the control, and decreased synaptophysin by 22% below the control. When added in HIV-infected MCM, His-CATB was internalized similarly, 2.72-fold, while cleaved caspase-3 increased 3.29-fold over the control, and synaptophysin dropped 25% below the control. Comparing these two treatments, there is not only more CATB internalization upon HIV-1 infection, but also a markedly greater level of apoptosis, likely caused by additional factors or processes triggered by HIV-infection not described by these experiments. His-CATB in uninfected MCM was 1.38-fold internalized over the untreated neurons, with same level of cleaved caspase-3 as the untreated neurons and a slight 7% decrease of synaptophysin below the untreated neurons, suggesting that protective factors in uninfected MCM decrease His-CATB internalization, apoptosis, and neuronal damage when compared to His-CATB exposure in neuronal culture media. CATB and SAPC antibodies decreased cleaved caspase-3, percentage of apoptosis and increased synaptophysin to basal levels. These results confirm the neuroprotection previously demonstrated by TUNEL experiments^6,7^. Although these results are important, we recognize that lack of statistical significance between the treatments suggests the existence of additional neurotoxins in the media, including viral proteins. Therefore, our experiments demonstrate that His-CATB is neurotoxic, in addition to other operative mechanisms occurring during HIV-infection. Other enzymes such as calpains, which are non-lysosomal cysteine proteases, could also contribute to HIV-induced neuronal damage through the murine double minute 4 (MDM4) degradation, a p53 regulator which is crucial for neuroprotection^23^. CATB interacts with matrix metalloprotease-9 (MMP-9) in uninfected MCM, an interaction that is not detected in HIV-infected MCM^7^. When neurons are exposed to HIV-infected MCM pre-treated with anti-MMP-9 antibodies, neurotoxicity remains high^7^. Therefore, MMP-9 is one of the MDM-secreted proteins that could be protecting neurons from damage in uninfected MCM. This data previously published by our group suggest that SK-N-SH exposed to uninfected MCM with His-CATB may exhibit lower levels of cleaved caspase-3 than SK-N-SH exposed to uninfected MCM (no His-CATB) because the addition of exogenous His-CATB promotes the interaction with more neuroprotective factors secreted by uninfected MDM. On the other hand, SK-N-SH exposed to uninfected and HIV-infected MCM (no His-CATB) may have high levels of cleaved caspase-3 due to pro-inflammatory factors secreted by activated MDM.

The exact mechanism that triggers the neuronal uptake of CATB remains to be elucidated. Possible ways for CATB entry to neurons are: (1) the permeabilization of the cellular membrane due to stress, (2) passive diffusion of the protein through the membrane or (3) active transport mechanisms. Viral protein gp120 utilizes dynamin-dependent endocytosis to enter neurons and trigger cell death^24^. Another fourth possible mechanism of neuronal entry that involves MCM, is the release of CATB from macrophages in EVs. Exosomes are small (less than 100 nm) extracellular vesicles containing miRNAs, proteins and lipids, secreted from one cell and internalized by another by membrane fusion^25–28^. During HIV infection, exosomes may transport a variety of cellular and viral factors, such as miRNAs and the viral protein Nef, modulating the recipient cell function, as reviewed recently^28^. Exosomes have been studied for their ability to facilitate HIV spread to neighboring cells^29^, for transferring microRNAs from HIV Tat-exposed astrocytes to neurons thus contributing to neuronal damage^26^, and transfer of amyloid peptides among other toxic and/or pro-inflammatory molecules as reviewed elsewhere^27,28,30^. Since CATB is an endogenous protein moving across the endolysosomal pathways, we tested whether exosomes were a possible pathway contributing to CATB secretion. Secretion of CATB and its partner SAPC was indeed detected in exosomes released from both uninfected and HIV-infected MDM, with higher concentration of CATB in exosomes secreted from HIV-infected MDM. However, purified exosome nor exosome-derived CATB/SAPC did not trigger the activity of caspase-3 but triggered a significant increase of DNA fragmentation in SK-N-SH exposed to HIV+ EVs, by TUNEL assay. These results suggest that EVs might trigger neuronal apoptosis through an alternate mechanism independent of caspase-3.

A possible fifth mechanism of internalization could be the presence of mannose-6-phosphate receptors (M6PR) in neurons. The M6PRs are involved in the movement of lysosomal proteases from the Golgi apparatus to the endoplasmic reticulum, the lysosome and the cellular membrane, facilitating intracellular compartment mobilization of proteins^17–19^. However, little is known about the expression of these receptors in neurons and their localization at the cellular membrane^31–35^. Several groups have reported increased expression of M6PR in the brain upon an inflammatory stimuli^36^, and in AD patients’ brains^37^. In addition, HIV uses these receptors to cross the BBB into the CNS^38^. In this study, we observed M6PR surface (8.1%) and intracellular (82.3%) expression in control SK-N-SH neurons by flow cytometry. Moreover, exposure of neurons to MCM increased the intracellular expression of M6PR in SK-N-SH, suggesting that factors secreted from 12 days cultured MDM alter M6PR expression in neurons. Future studies are intended to determine if exposure to HIV-infected MCM recruits more M6PR to the neuronal surface, compared to uninfected and untreated cells.

In line with the numerous factors secreted by activated MDM upon HIV infection, additional factors might compete with CATB for a neuronal receptor as an uptake mechanism. This could explain why in MCM from some HIV-infected donors the CATB is internalized at lower levels than in the uninfected counterparts. The exact mechanism by which macrophage-secreted CATB triggers neuronal dysfunction remains unknown. What we know from the literature is that CATB triggers caspase apoptotic pathways directly and as a downstream mediator, with TNF-α inducing its translocation from the lysosomes to the cytosol^8,39–43^. In addition, CATB promotes the release of cytochrome c and mitochondrial mediated apoptosis^41,44,45^. We demonstrate in this study that HIV-infected MCM increases neuronal internalization of CATB (assessed by His-CATB), effects caspase-3/7 activity (without the addition of His-CATB) tending to be higher than uninfected controls (p = 0.0787), and is reduced by CATB antibodies, SAPC antibodies, and CA074. Therefore, CATB-induced neuronal dysfunction is enhanced by HIV-infected MCM and involves apoptotic pathways that can be reduced *in vitro*. Future studies must continue to determine if CATB -induced neurotoxicity can be reduced *in vivo* with a small HIV-animal model.

Another mechanism of neurotoxicity is amyloid peptide aggregation, which is the predominant mechanism of neuronal dysfunction in Alzheimer’s disease (AD) patients^46–52^. HIV patients can also develop Aβ plaques^53–57^. CATB has been implicated in the formation, aggregation and degradation of Aβ peptides in AD, in contrasting studies^58–62^. It has been demonstrated in mice models of AD, that CATB inhibitors improve memory and reduce Aβ plaques in the brain^60,63–67^. CATB is also increased in plasma of AD patients^68^. SAPC, which interacts with CATB in HIV-infected MCM but not in uninfected controls, has been also implicated in amyloidosis^69^, the stabilization of amyloid fibrils^70^ in the brain and is related to dementia in AD patients^71^. SAPC is also a neurotoxic protein *in vivo* when injected to rats’ brains^72^ or in HIV-infected MCM added to neurons *in vitro*^7^. In this study, Aβ peptides levels significantly increased in SK-N-SH exposed to HIV-infected MCM. Moreover, the quantification of the post-mortem deep white matter frontal cortex tissues revealed that CATB and Aβ_1–42_ peptides are higher in patients with cognitive impairment. The inhibition of CATB and SAPC in HIV-infected MCM before exposure to rat cortical neurons and consequent reduction in Aβ and cleaved caspase-3, suggests that the CATB/SAPC complex participates in the formation of Aβ_1–42_ peptides and inhibition of this complex can decrease these neurotoxic aggregates, emphasizing the potential of targeting the CATB/SAPC complex in HIV infection. On the other hand, exogenous addition of His-CATB decreased Aβ load in SK-N-SH to basal levels (compared to untreated control), opposed to SK-N-SH exposed to uninfected and HIV-infected MCM (without His-CATB). This finding could explain why CATB increases upon HIV infection. While infusing the culture media with exogenous His-CATB leads to neurotoxicity, increasing proteolysis in the environment might be forcing the degradation of Aβ peptides. Currently, the relationship between CATB and Aβ in the literature remains unclear: Does CATB promote Aβ clearance or accumulation upon HIV infection? We have demonstrated that in HIV-infected MDM, cystatins no longer inhibit CATB. In turn, it decreases the interferon response and promotes HIV replication^73^. Meanwhile, dysregulated CATB is secreted from MDM^6^, with different extracellular protein-protein interactions^7^. We hypothesize that HIV infection triggers the accumulation of Aβ plaques, increases the expression and secretion of dysregulated CATB and interacting partners, together with viral toxins and pro-inflammatory factors, activating the overall proteolytic activity in the environment, ultimately contributing to neurocognitive impairment. In this study, we confirmed that HIV infection promotes an increase in Aβ levels in neurons and that targeting altered CATB protein complexes such as CATB/SAPC with specific antibodies is neuroprotective. Recently, it was demonstrated that HIV-infected MCM increases the beta-secretase 1-site amyloid precursor protein cleaving enzyme-1 (BACE1) in rat neurons, leading to neuronal loss, which could also be prevented by targeting the enzyme^74^. Furthermore, this group reported increased BACE1 expression in the hippocampus of post-mortem brain tissues from HIV-seropositive patients. Perhaps both enzymes are activated by HIV infection of macrophages, increasing Aβ formation and neurotoxicity. From our experiments, we cannot conclude if His-CATB-induced Aβ decrease is due to interaction of His-CATB directly with Aβ peptides or indirectly through activation of other enzymes and subsequent increased proteolytic activity. Thus, more studies are needed to elucidate the specific role of CATB in Aβ plaque formation/clearance, how the changes in protein-protein interactions differently alters this process, and how it affects the HIV-induced Aβ accumulation in the brain. In future studies, we will determine the role of MDM-derived CATB in BACE1 expression.

MDM inter-donor variability in the response to HIV infection is a limitation in the search for specific mechanisms and in signaling studies. Therefore, CATB-induced neuronal apoptosis using cell lines and animal models will better serve to elucidate these mechanisms. Recent studies with the HIVE mice demonstrated that CATB was increased in the striatum^75^. Because of the diversity of results obtained from different MDM donors and the slight changes upon treatments, we could hypothesize that the mechanism of CATB-induced neuronal injury might differ from one patient to another. In addition, since CATB is involved in several functions at the intracellular and extracellular levels, it is possible that CATB is using a combination of these different mechanisms instead of driving the response with one pathway.

In conclusion, CATB and SAPC secreted from HIV+ macrophages might induce neuronal dysfunction and death by three different mechanisms: (1) CATB internalization, (2) activation of apoptotic cascade(s) converging in the cleavage of caspase-3 and (3) triggering the aggregation of Aβ peptides. The interplay between these mechanisms is affected by the levels of infection and the inherent differences in the immunological response of the macrophage donors, which was beyond the scope of this study. CATB/SAPC interaction represents a novel target for the development of therapies against HIV-associated neuronal dysfunction in HAND patients.

## Methods

### Isolation and infection of monocyte-derived macrophages from HIV seronegative donors

Blood was collected as part of the NIH/NIGMS SC1 project entitled: “Targeting monocyte/macrophage CATB interactome in HIV-1 neurocognitive disorders”, with approval from the University of Puerto Rico Medical Sciences Campus Institutional Review Board, Human Research Subjects Protection Office (Protocol 0720116) and by obtaining written informed consents from the participants. All the experiments were conducted in accordance with the institutional guidelines and regulations. Peripheral blood mononuclear cells (PBMC) were isolated from the blood of six healthy women by Ficoll® gradient centrifugation (MP Biomedicals, Santa Ana, CA), following manufacturer’s instructions. MDM were selected by adherence and inoculated with HIV-1_ADA_ at a 0.1 MOI^6^. Supernatants were collected at 3, 6, 9 and 11 days post-infection (dpi) to assess the infection levels of HIV-p24 antigen concentration by ELISA, following manufacturer’s instructions (XpressBio, Frederick, MD). At 11dpi, the media was exchanged for serum-free RPMI-1640. After 24 hours of incubation, serum-free 12dpi MDM supernatants were collected and centrifuged to remove cell debris, then used or stored at −80 °C until use. Serum-free 12dpi MDM supernatants are termed macrophage-conditioned media (MCM) for the rest of this study. MCM was diluted 1:4 in EMEM for neurotoxicity assays.

### SK-N-SH culture and lysate preparation

SK-N-SH (HTB-11; ATCC) human neuroblastoma cells were cultured in Eagle’s Minimal Essential Medium (EMEM) supplemented with 10% FBS, 1% sodium pyruvate, 1% non-essential amino acids and 1% penicillin/streptomycin. Cells were cultured at 37 °C, 5% CO_2_ until more than 75% confluence for subculture. For activation of apoptotic cascades or CATB uptake, cells were cultured in 6-well plates at a density of 5 × 10^5^ cells/well. Neurons were exposed to His-CATB for 24 hours, diluted in serum-free EMEM, alone or with the following pre-treatments: anti-CATB antibodies (Sigma-Aldrich #C6243, final concentration of [12.5 µg/mL]), anti-SAPC antibodies (Abcam #ab87914, final concentration of [14.1 µg/mL]) or CA074 (Sigma-Aldrich #C5732, final concentration of [10 µM]). After the incubation, cells were washed and detached on ice using RIPA buffer (Abcam, Cambridge, UK) with 5% protease inhibitor cocktail without cysteine proteases inhibitors and centrifuged at 4 °C for 30 minutes at 12,000 rpm. Supernatants containing the lysate proteins were stored at −80 °C until use. Data represents a minimum of three technical replicates for all treatments.

### Intracellular flow cytometry

Active recombinant histidine-tagged CATB (His-CATB; Sino Biological) was diluted to a concentration of 250 ng/mL in MCM from MDM control or infected with HIV-1_ADA_ (0.1 MOI) up to 12 days (n = 6 MDM donors). His-CATB concentration was selected based on previous CATB concentration measurements in uninfected and HIV-infected MCM by ELISA. SK-N-SH neuroblastoma cells cultured in 6-well plates at 5 × 10^5^ cells/well density were exposed to His-CATB for 24 hours. Then the cells were detached and stained with phycoerythrin (PE)-conjugated anti-Histidine tagged antibody (10 uL/10^6^ cells: R&D systems, Minneapolis, MN; # IC050P) using 3% FBS in PBS as stain buffer and BD transcription factor buffer flow cytometry set, following manufacturer’s instructions. IgG antibody isotype stained and unstained neurons were used as negative controls. Acquisition of % PE-positive neurons was performed in a FACSCalibur flow cytometer (Becton Dickinson, Franklin Lakes, NJ). For the surface and intracellular staining of mannose-6-phosphate receptor, we used a mouse monoclonal anti-M6PR conjugated to Alexa Fluor® 488 (Abcam, #ab205812).

### Western blots

Protein concentration was measured using a DC assay^6^, and 10–30 µg of total protein from neuronal lysates, exosomes, or MDM lysates were loaded into 4–20% TGX gels (Bio-Rad, Hercules, CA). PVDF membranes were probed with: rabbit polyclonal anti-cleaved caspase-3 (1:250, Cell Signaling Technology, Danvers, MA; #9661 s), mouse monoclonal anti-GAPDH (1:500, Santa Cruz Biotechnology, Dallas, TX; #sc-365062), rabbit monoclonal anti-synaptophysin (1:500, Abcam #ab32127), rabbit polyclonal anti-CD81 (1:500, System Biosciences, Palo Alto, CA; ExoAb-Kit-1), rabbit polyclonal anti-heat shock protein 70 (Hsp70, 1:500, System Biosciences ExoAb-Kit-1), and rabbit polyclonal anti-CD63 (1:500, System Biosciences ExoAb-Kit-1), incubated overnight at 4 °C. HRP-conjugated goat anti-mouse or anti-rabbit secondary antibodies (1:15,000; Sigma-Aldrich) were incubated for 1 hour. Images were acquired and analyzed using a Gel Doc XR+ equipped with ImageLab™ software (Bio-Rad). Densitometry was performed by dividing the volume (intensity) of each protein by the volume intensity of the GAPDH band per lane, per membrane. Membranes were re-probed incubating in SDS/glycine mild stripping buffer (pH 2.2) for 20 min followed by washes with PBS and TTBS, and then blocking and probing with the next antibody of interest. Data represents the results of Western blots in duplicates for all treatments, presented as mean ± SEM.

### Caspase-3/7 activity assay

Caspase-3/7 activity was measured *in situ* in neurons cultured in 96-well plates at density of 2 × 10^4^ cells/well, using the Caspase-Glo® 3/7 luminometry assay (Promega, Madison, WI), following manufacturer’s instructions. Briefly, SK-N-SH were incubated with MCM from uninfected or HIV-infected MCM (n = 3) pre-treated with anti-CATB antibodies, anti-SAPC antibodies or CA074 cathepsin B inhibitor for 24 hours at 37 °C, 5% CO_2_. Then caspase-3/7 substrate was added in substrate buffer and incubated for 2 hours in the dark at room temperature. Luminescence was measured in a Varioskan Flash plate reader (Thermo Scientific, Waltham, MA). Relative luminescence units (RLU) were measured in duplicate for each condition and each donor after blank subtraction. Data reported in fold change over the control, untreated neurons for this experiment, as mean ± SEM.

### Exosome isolation from MCM

Uninfected and HIV-infected MCM from day 12 post-infection (n = 4 MDM donors) was centrifuged at 2,000 × g for 30 minutes to remove cell debris. A volume of 1 mL of MCM was mixed with 0.5 mL of Total Exosome Isolation Reagent (Invitrogen™, Carlsbad, CA), and incubated overnight at 4 °C. The next day, samples were centrifuged at 10,000 × g for 1 hour at 4 °C. The supernatant was collected and termed exosome-depleted fraction. The pellet containing EVs was resuspended in sterile filtered PBS and stored at −80 °C for protein quantification, nanoparticle tracking analysis (NTA; System Biosciences custom service) and western blot analyses. NTA analysis results were added in the Supplementary Information (Supplementary Fig. 5).

### Quantification of apoptotic cells by TUNEL assay

SK-N-SH were exposed to His-CATB [250 ng/mL] and all the treatments described above, diluted in plain EMEM. Cells were treated and incubated at 37 °C for 24 hours as described^6^. His-CATB/EMEM solution was pretreated with specific cathepsin B inhibitor CA-074 (Sigma-Aldrich, 10 µM) or monoclonal anti-cathepsin B, or anti-SAPC antibodies. During apoptosis, fragmented DNA exhibits green fluorescence upon TUNEL labeling. A minimum of three images were acquired for each condition for each donor. Green fluorescent nuclei were counted using ImageJ software (NIH) and divided by the total number of neurons (all DAPI-positive nuclei, blue), to obtain a percentage of apoptotic neurons. A dilution of 30% exosomes in EMEM and 25% MCM in EMEM were added. Negative controls included SK-N-SH exposed to His-CATB solution pre-treated with IgG1 isotype control, His-GAPDH [250 ng/mL] (as a Histidine-tagged protein negative control) added in EMEM, uninfected MCM and HIV + MCM, and untreated SK-N-SH incubated in plain EMEM. TUNEL assay positive control constitutes SK-N-SH treated with DNaseI to promote DNA fragmentation and positive green staining.

### Immunofluorescence of post-mortem brain tissue

Post-mortem brain tissue samples were provided by National NeuroAIDS Tissue Consortium (NNTC) or the Alzheimer’s Disease Research Center (ADRC) and processed as described previously^22^. Double immunofluorescence labeling of paraffin-embedded deep frontal white matter tissue samples from healthy subjects (n = 3), patients diagnosed with HIV-infection with normal cognition (n = 3) or with HIV-associated neurocognitive impairment (n = 4) and AD patients (n = 2). The methods, antibodies and pictures of these tissues were published in^7^, but were not quantified. Images were acquired using a Nikon Eclipse E400 fluorescence microscope with a SPOT Insight QE camera and SPOT 5.1 software. At least three images were acquired from each section. Fluorescence was measured and reported as integrated density in function of the area measured, using ImageJ (NIH) software. Reported relative fluorescence was normalized against the number of cells per field and the fluorescence/cell measured in the negative controls.

### Isolation and culture of human primary neurons

As previously reported, human fetal brain tissue (16–18 weeks, gestation) was obtained from elective abortion procedures performed in full compliance with National Institutes of Health and Temple University ethical guidelines. The tissue was washed with cold Hanks balanced salt solution (HBSS), and meninges and blood vessels were removed. The tissue was digested with papain (20 mg/ml) for 30 min at 37 °C for isolation neurons. The digestion was neutralized with fetal bovine serum (FBS), and the tissue was further dissociated to obtain single-cell suspensions. After obtaining a single-cell suspension, cells were plated at a density of ~1.8 × 10^6^ cells/60 mm dish coated with poly-D lysine in neurobasal media with B27 supplement, horse serum, and gentamicin (NM5). After ~2 h, media was removed neurobasal media was added. Cultures were re-fed 24 h later with a complete change of neurobasal media without horse serum (NM0). After 4 days in culture, 1/4 of the media was removed and replaced with NM0 supplemented with FDU and uridine. Purity of specific neurons was assessed by immunolabeling with anti-MAP2/neurofilament for neurons^76,77^.

### Statistical analyses

His-CATB concentration dependent neurotoxicity curve was analyzed using Pearson correlation. The percentage of PE-positive neurons, and western blot densitometry for neurons exposed to His-CATB in plain media with different pre-treatments and in MCM, were analyzed by One-way ANOVA and Tukey’s multiple comparisons. The percentage of PE-positive neurons obtained by Western blots after incubating neurons with MCM and EVs were analyzed using paired t-tests. Spearman correlation was performed to compare the change (HIV+ minus Uninfected) in percentage of PE-positive neurons (∆PE+ neurons) with the log_10_ of the HIV-p24 concentration. Caspase-3/7 activity of neurons exposed to MCM was analyzed using repeated measures two-way ANOVA with Tukey’s multiple comparison’s test. P-values less than 0.05 were considered statistically significant. All analyses were conducted in Prism® GraphPad software 6.01.

## Supplementary information

HIV Infection Induces Extracellular Cathepsin B Uptake and Damage to Neurons supplementary information

## Data Availability

Most of the data generated or analyzed during this study are included in this published article (and its Supplementary Information file). All datasets generated during and/or analyzed in the current study are available from the corresponding author on reasonable request.
