# Supplementary material for: HIV Infection Induces Extracellular Cathepsin B Uptake and Damage to Neurons
Source: Sci Rep. 2019 May 29;9:8006. doi: 10.1038/s41598-019-44463-1 (PMC6541605; doi:10.1038/s41598-019-44463-1)

HIV Infection Induces Extracellular Cathepsin B Uptake and Damage to Neurons

Yisel M. Cantres-Rosario[1], Sarah C. Ortiz-Rodríguez[1], Aemil Santos[2], Marines Plaud[1], Karla Negron[2], Bianca Cotto[3], Dianne Langford[3] and Loyda M. Melendez[1*]

Supplementary Information

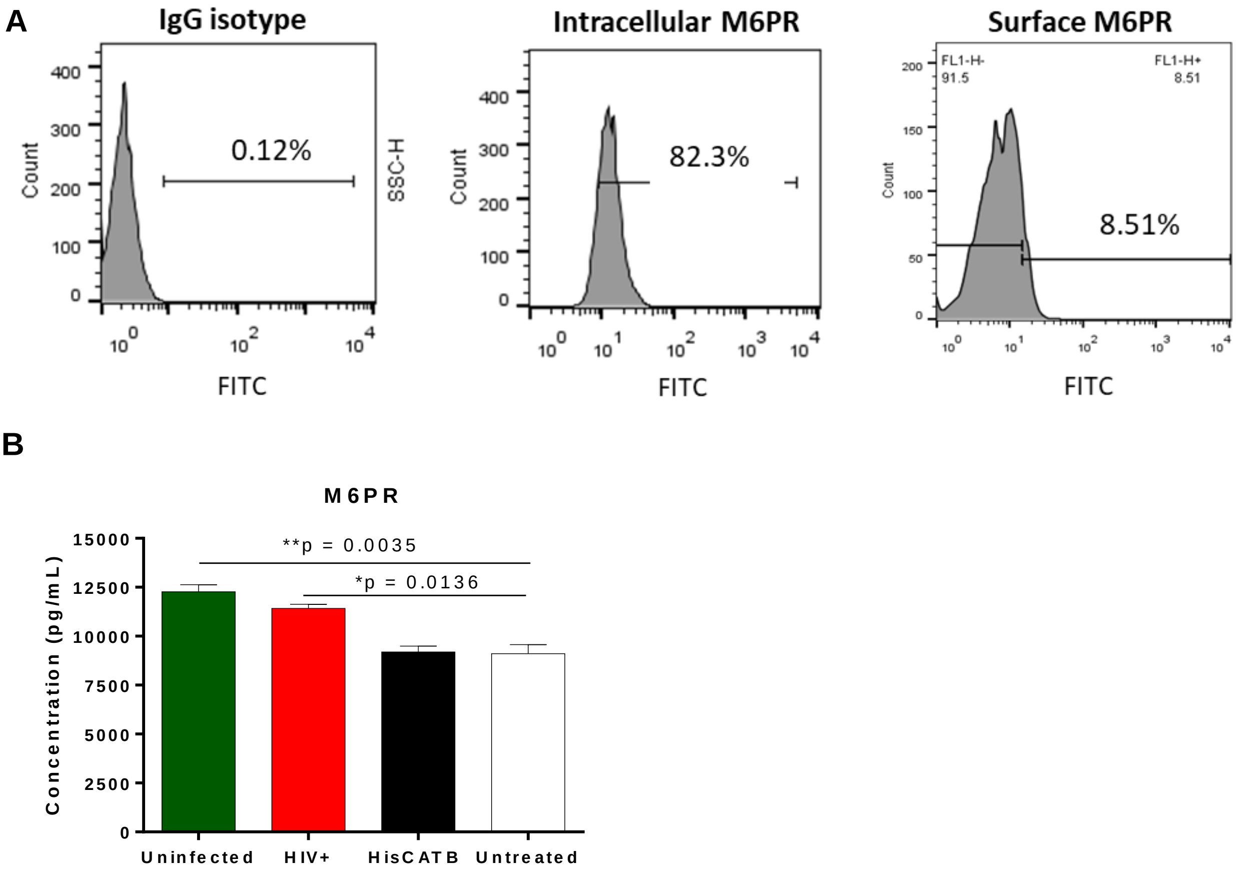

Supplementary Figure 1. Expression of the mannose-6 phosphate receptor in SK-N-SH by flow cytometry. (A) Neurons were detached and stained for the expression of the M6PR at intracellular and surface levels, in comparison to neurons stained with an IgG isotype. Percentages of positive neurons were acquired in a FACSCalibur flow cytometer (Becton Dickinson, Franklin Lakes, NJ). (B) SK-N-SH were exposed to 12dpi uninfected or HIV-infected MCM, and His-CATB (250ng/mL). M6PR levels were measured in the whole cell lysates by sandwich ELISA and analyzed by four-parameter logistic. Experiment performed in duplicates and presented as mean ± SEM.

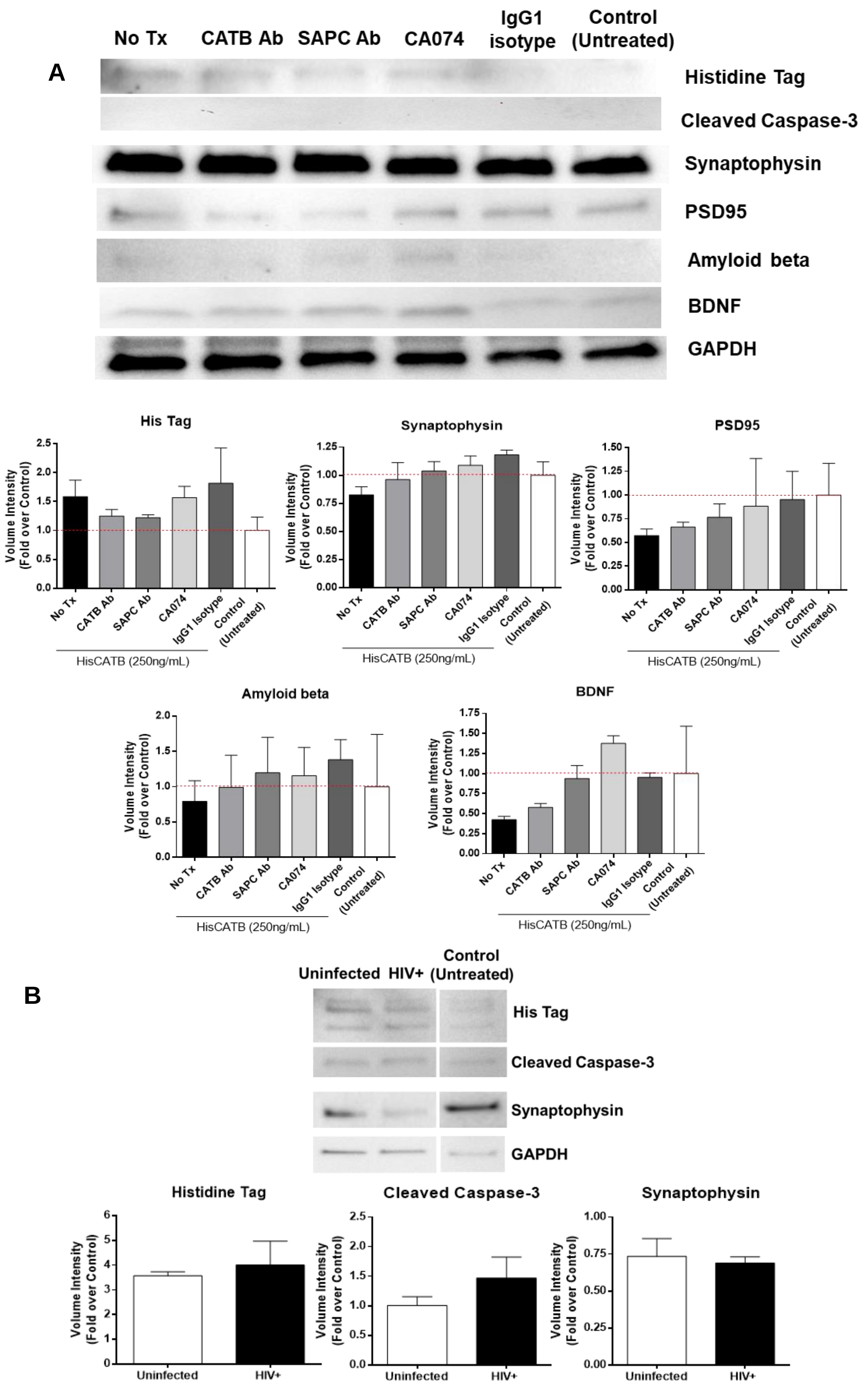
A
No Tx
CATB Ab
SAPC Ab
CA074
IgG1 isotype
Control (Untreated)
Histidine Tag
Cleaved Caspase-3
Synaptophysin
PSD95
Amyloid beta
BDNF
GAPDH
His Tag
Synaptophysin
PSD95
Amyloid beta
BDNF
Volume Intensity (Fold over Control)
IgG1 Isotype
HisCATB (250ng/mL)
B
Uninfected
HIV+
Control (Untreated)
His Tag
Cleaved Caspase-3
Synaptophysin
GAPDH
Histidine Tag
Cleaved Caspase-3
Synaptophysin
Volume Intensity (Fold over Control)
Uninfected
HIV+

Supplementary Figure 2. Primary neurons exposed to His-CATB in macrophage conditioned-media. (A) Primary human neurons were exposed to His-CATB (250ng/mL) in culture media alone or in presence of His-CATB 250ng/mL alone (No Tx), or pre-treated with cathepsin B antibody (CATB Ab), SAPC antibody (SAPC Ab), CA074, or IgG1 isotype as negative control. Neurons were lysed and compared by western blot. Densitometry analyses for volume intensity normalized against GAPDH of histidine tagged cathepsin B, cleaved caspase-3, amyloid beta, BDNF, PSD95 and synaptophysin, expressed as fold over the control (untreated neurons) for comparison. Data presented as mean ± SEM of n=2 experiments. (B) Primary neurons isolated from the cortex of E18 rat pups were exposed to His-CATB in Neurobasal media alone, uninfected or HIV-infected MCM for 24 hours. Neuronal lysates were assessed for His-CATB internalization, activation of caspase-3, and synaptophysin levels by western blot. Data expressed in Mean ± SEM. Western blotting performed in duplicate.

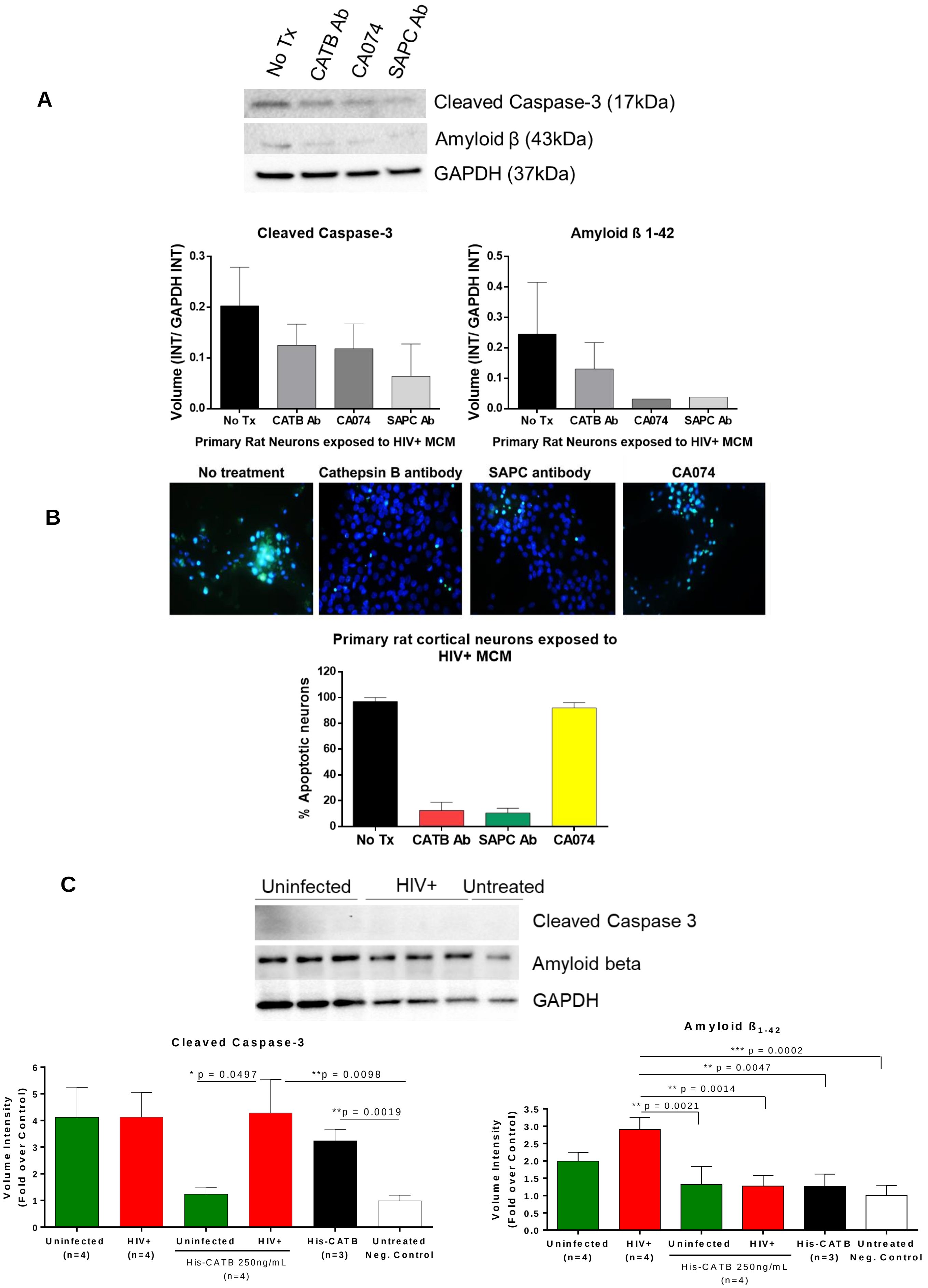
A
No Tx
CATB Ab
CA074
SAPC Ab
Cleaved Caspase-3 (17kDa)
Amyloid β (43kDa)
GAPDH (37kDa)
Cleaved Caspase-3
Volume (INT/ GAPDH INT)
0.3
0.2
0.1
0.0
No Tx
CATB Ab
CA074
SAPC Ab
Primary Rat Neurons exposed to HIV+ MCM
Amyloid ß 1-42
Volume (INT/ GAPDH INT)
0.5
0.4
0.3
0.2
0.1
0.0
No Tx
CATB Ab
CA074
SAPC Ab
Primary Rat Neurons exposed to HIV+ MCM
B
No treatment
Cathepsin B antibody
SAPC antibody
CA074
Primary rat cortical neurons exposed to HIV+ MCM
% Apoptotic neurons
120
100
80
60
40
20
0
No Tx
CATB Ab
SAPC Ab
CA074
C
Uninfected
HIV+
Untreated
Cleaved Caspase 3
Amyloid beta
GAPDH
Cleaved Caspase-3
Volume Intensity (Fold over Control)
6
5
4
3
2
1
0
* p = 0.0497
**p = 0.0098
**p = 0.0019
Uninfected (n=4)
HIV+ (n=4)
Uninfected
HIV+
His-CATB 250ng/mL (n=4)
His-CATB (n=3)
Untreated Neg. Control
Amyloid ß1-42
*** p = 0.0002
** p = 0.0047
** p = 0.0014
** p = 0.0021
Volume Intensity (Fold over Control)
3.5
3.0
2.5
2.0
1.5
1.0
0.5
0.0
Uninfected (n=4)
HIV+ (n=4)
Uninfected
HIV+
His-CATB 250ng/mL (n=4)
His-CATB (n=3)
Untreated Neg. Control

Supplementary Figure 3. Apoptosis and amyloid β_{1-42} peptides in neurons exposed to macrophage-conditioned media. (A) Primary rat cortical neurons were exposed to HIV-infected MCM alone (No Tx), or pre-treated with anti-CATB antibodies (CATB Ab), cathepsin B inhibitor CA074 or anti-SAPC antibodies (SAPC Ab). Neuronal lysates were assessed for cleaved caspase-3 as a marker of apoptosis and amyloid β_{1-42} peptides levels by western blot. Densitometry was performed normalizing cleaved caspase-3 and amyloid β_{1-42} peptides volume intensity against the volume intensity of GAPDH. Western blotting was performed in duplicate. (B) Primary rat cortical neurons were cultured in glass chamber-slides and exposed to HIV-infected MCM alone (No Tx) or with anti-CATB antibodies, anti-SAPC antibodies or CA074 inhibitor, for 24 hours. Cells were fixed and stained with in situ cell death fluorescein kit (TUNEL assay). A minimum of three pictures per treatment were acquired by fluorescence microscopy. Green labeled nuclei are markers of apoptosis, and were quantified using ImageJ software. The graph represents the mean ± SEM of percentage of apoptotic neurons (green over blue nuclei ratio). (C) SK-N-SH were exposed to uninfected and HIV-infected MCM with or without His-CATB, and to His-CATB in culture media. Whole cell lysates were collected and levels of cleaved caspase 3 and amyloid beta were measured by western blot. Western blot picture represents whole cell lysates of SK-N-SH exposed to uninfected and HIV+ MCM from n=3 different MDM donors (without His-CATB). Western blot results of SK-N-SH exposed to His-CATB are presented in Fig. 4.

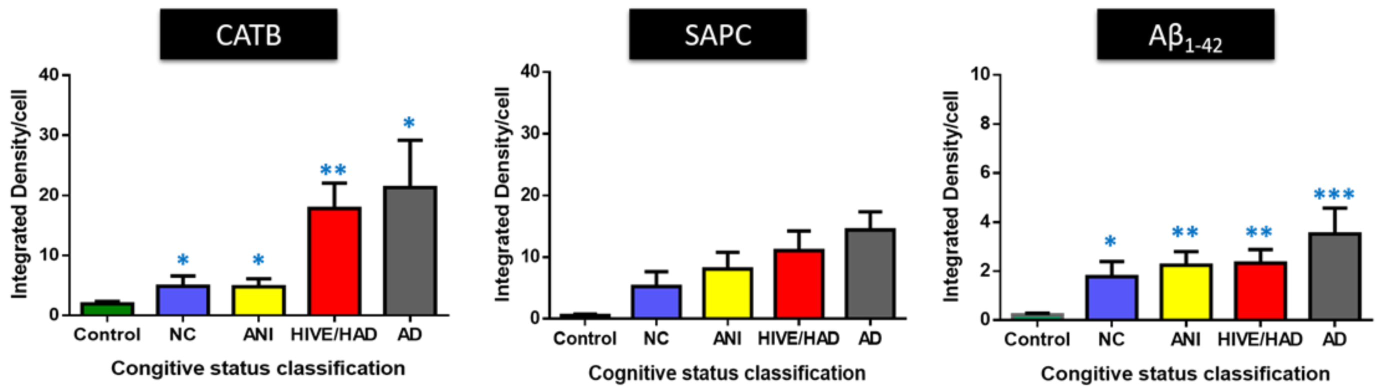

Supplementary Figure 4. Quantification of immunofluorescence performed in deep frontal white matter post-mortem brain tissue from HIV-infected and Alzheimer's patients. Immunofluorescence staining of paraffin-embedded post-mortem brain tissue samples and quantification was performed in three individuals from each HAND category and controls (n=3/group; N=12) and two AD patients. Expression of cathepsin B, SAPC and Aβ1-42 from brain tissue samples reported previously (Cantres-Rosario et al., 2015) was quantified recently using ImageJ (NIH) software, from a minimum of three pictures per tissue. Reported relative fluorescence was normalized against the number of cells per field and the fluorescence/cell measured in the negative controls. Statistical significance was established as *$p < 0.05$, **$p < 0.01$ and ***$p < 0.005$.

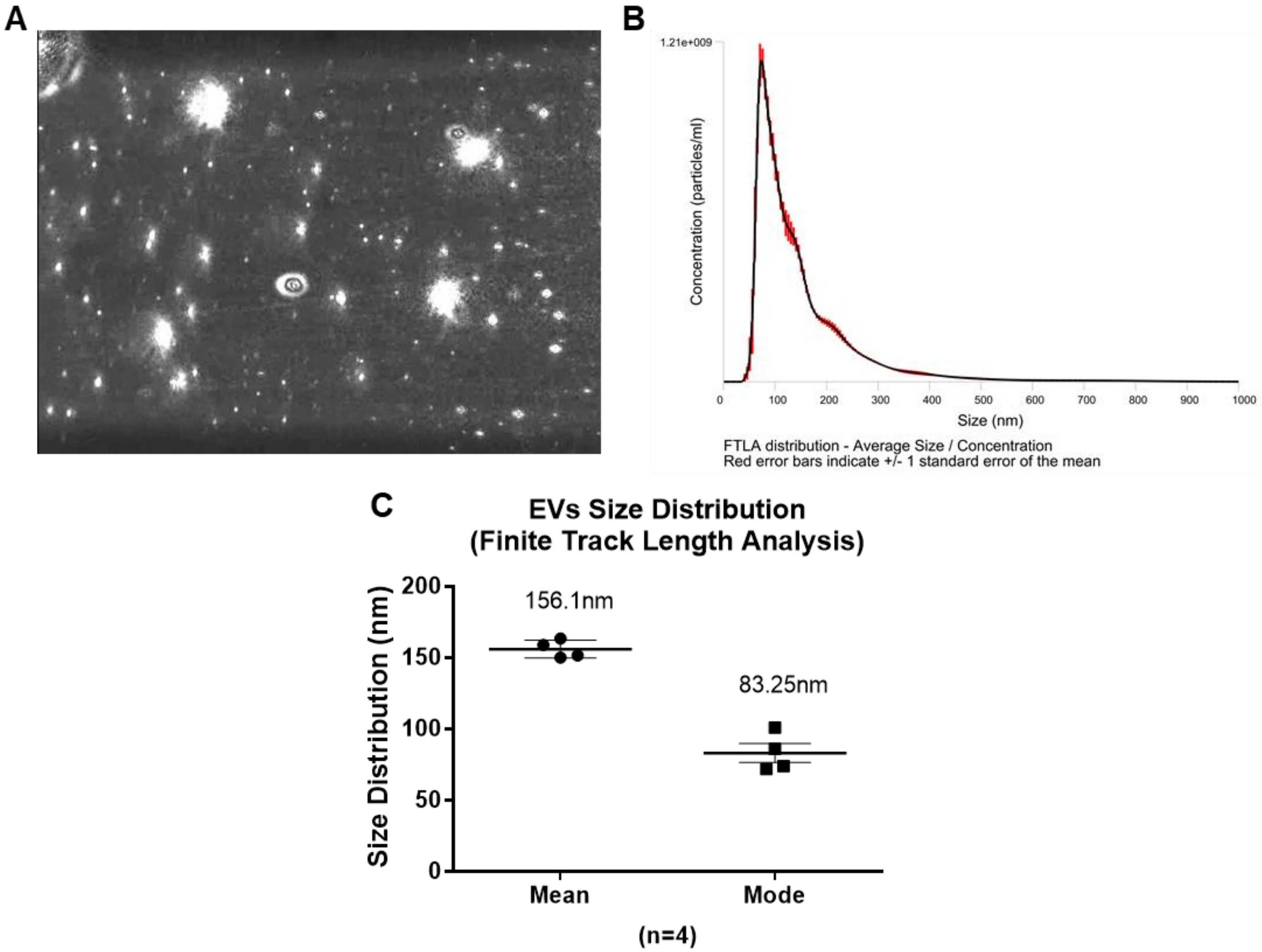

Supplementary Figure 5. Nanoparticle Tracking Analysis of extracellular vesicles derived from macrophage-conditioned media. (A) Representative picture of a preparation of extracellular vesicles (EVs) derived from MCM from one donor. EVs were isolated from MCM derived from four MDM donors, and analyzed by the System Biosciences Company. (B) Representative graph of the size distribution of the EVs from one donor, by finite track length analysis. (C) Mean and mode of the EVs size distribution (nm) of the four samples analyzed for exosome characterization.

Supplementary Information part 2: Complete membrane pictures for western blot figures

FIGURE 2A

FIGURE 2: Histidine Tag (25, 30 and 40kDa)

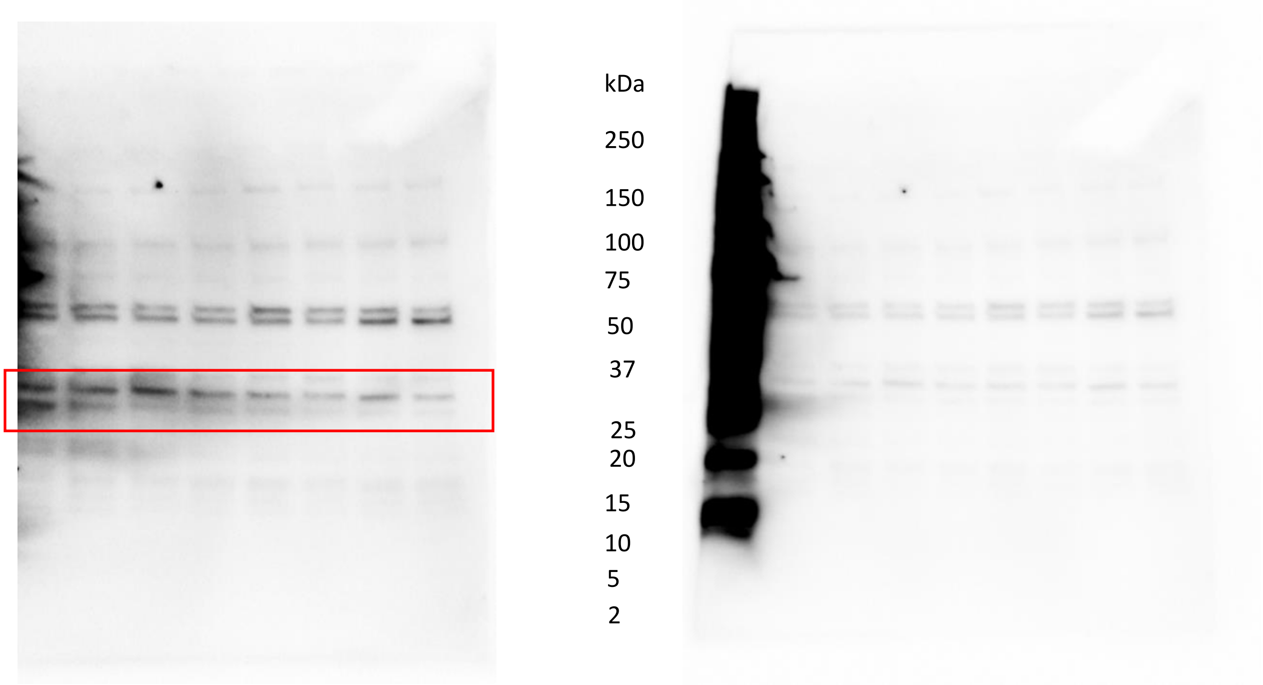

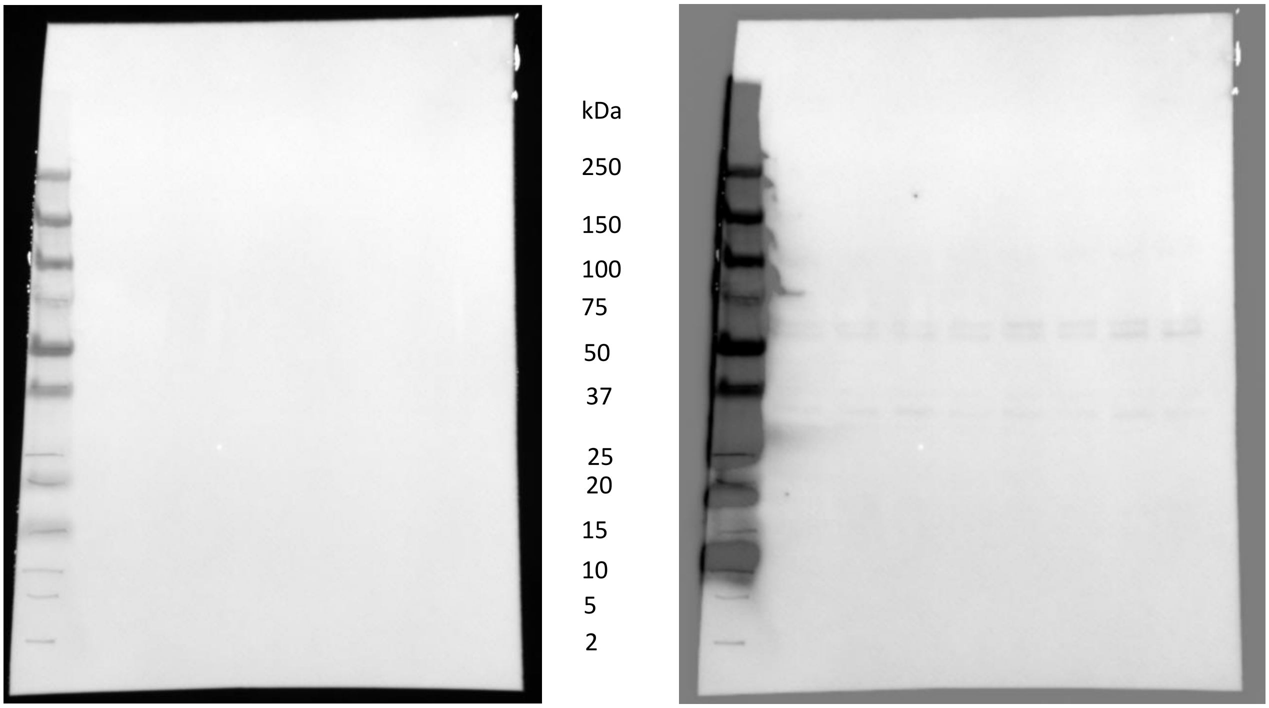

FIGURE 2: Cleaved Caspase 3 (17 and 19kDa)

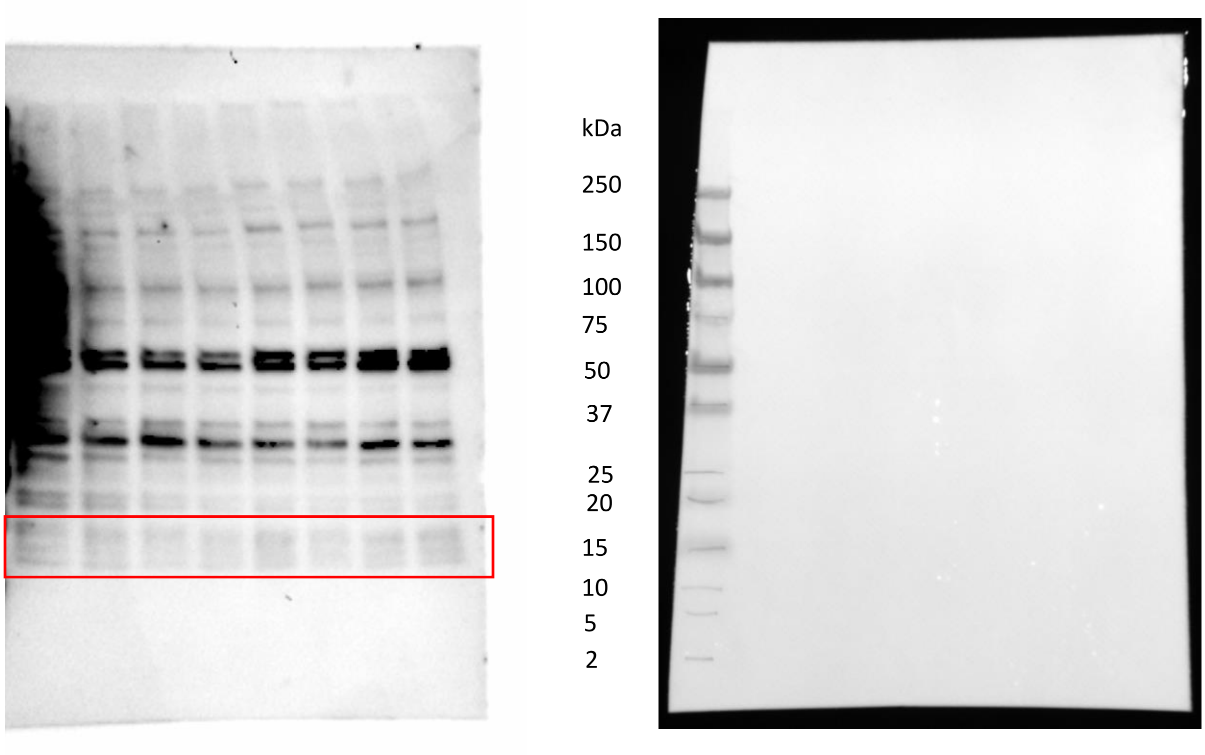

FIGURE 2: Synaptophysin (38kDa)

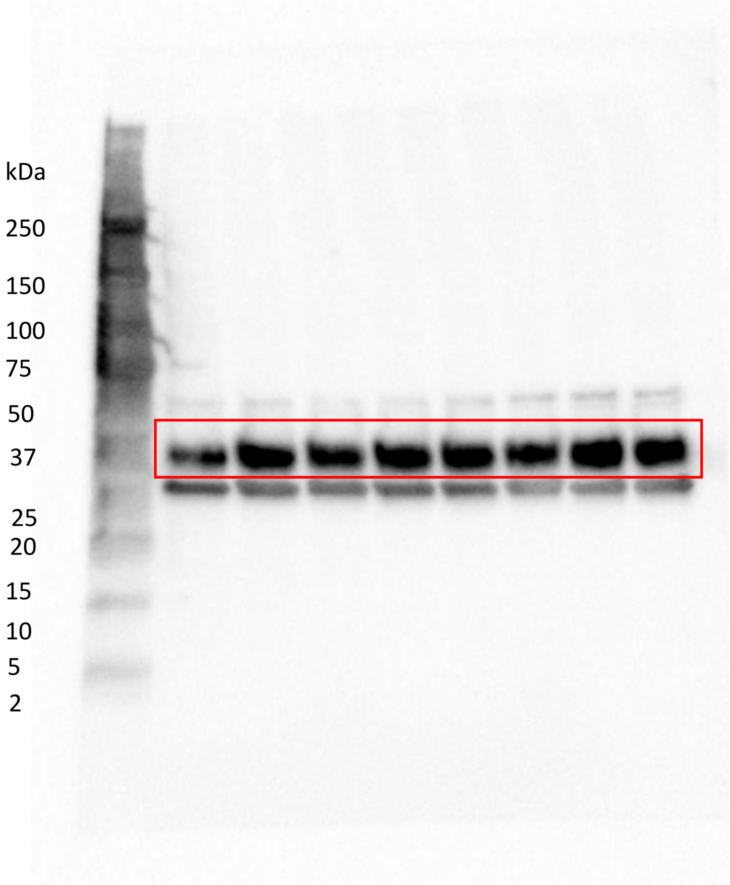

FIGURE 2: Amyloid beta (oligomeric; 50-75kDa)

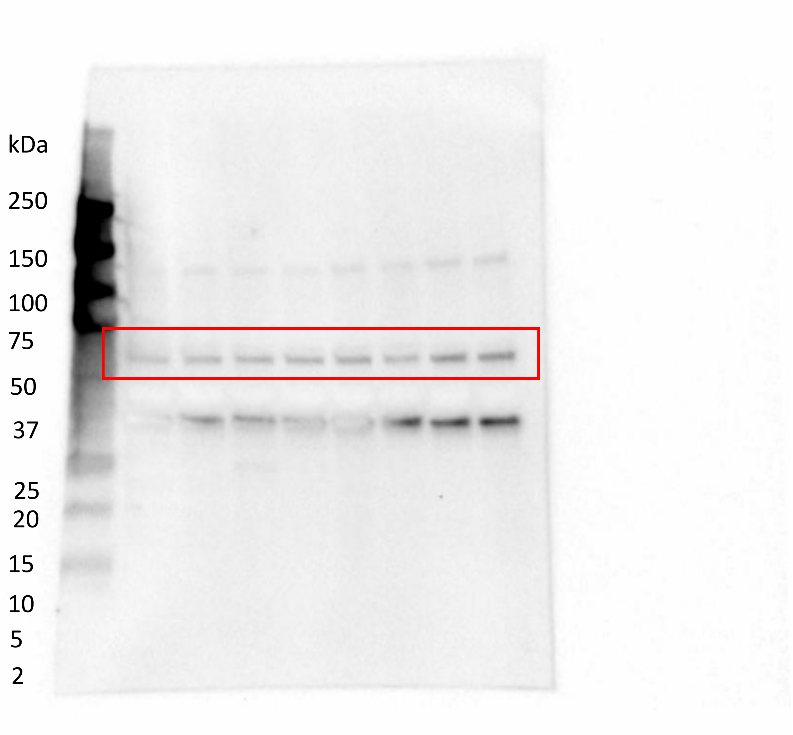

FIGURE 2: GAPDH (37kDa)

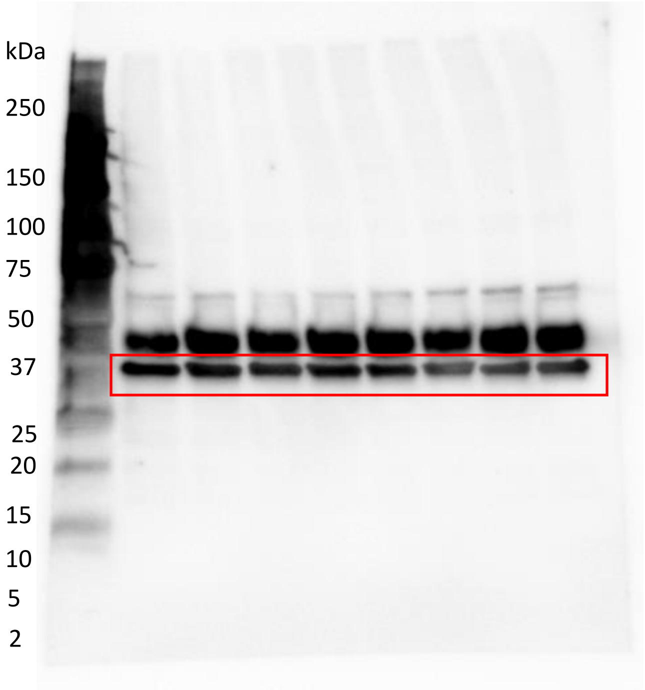

FIGURE 4A

FIGURE 4: Histidine Tag (Cathepsin B molecular weight: 25, 30 and 40kDa)

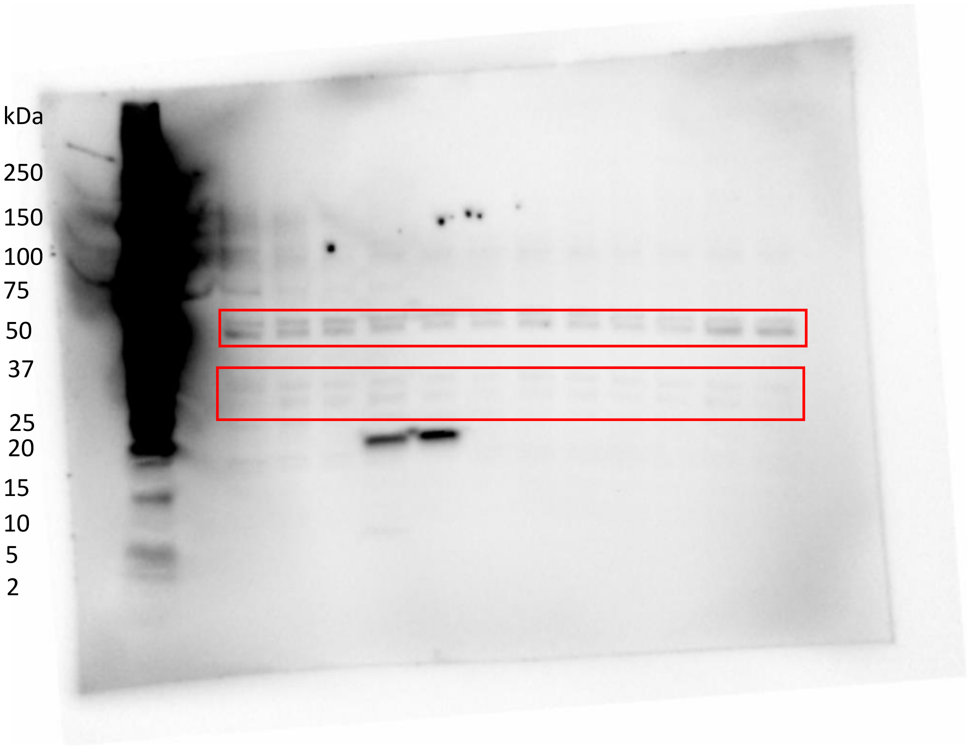

FIGURE 4: Cleaved Caspase 3 (17 and 19kDa)

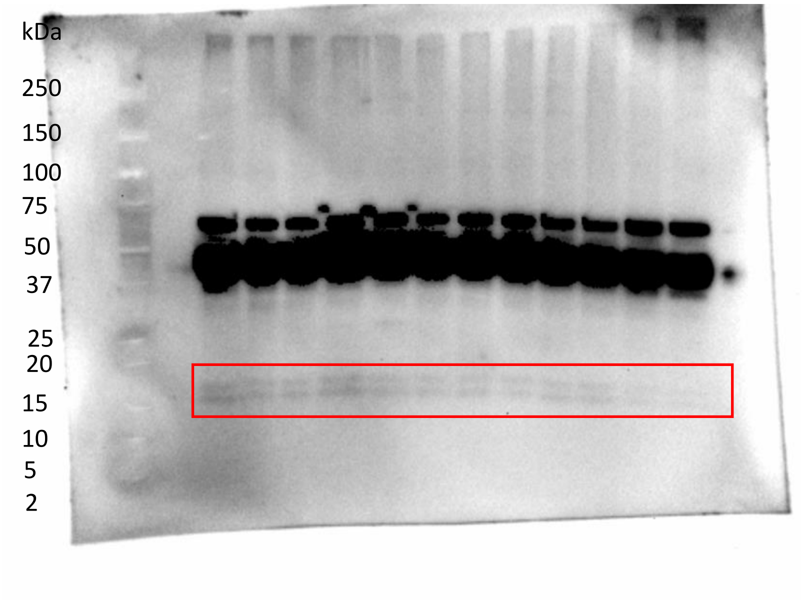

FIGURE 4: Synaptophysin (38kDa)

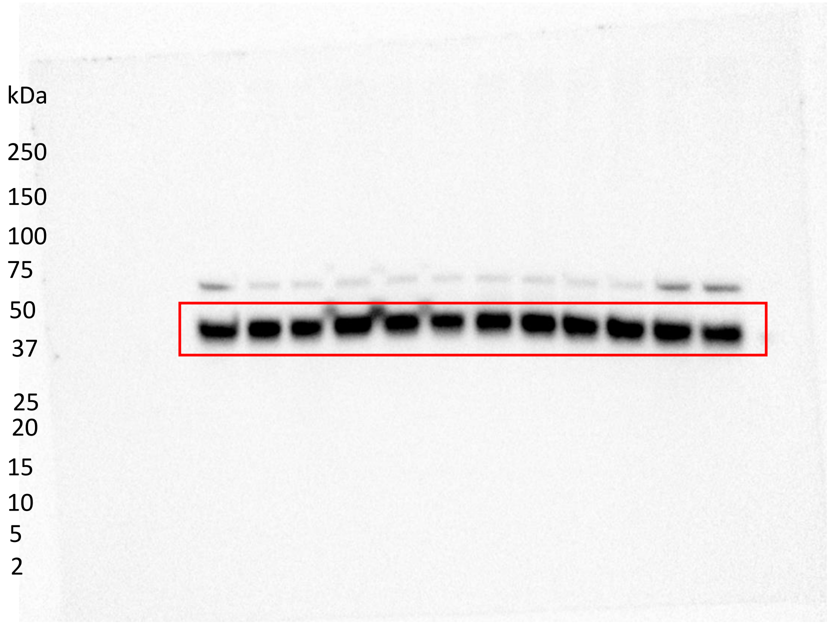

FIGURE 4: GAPDH (37kDa)

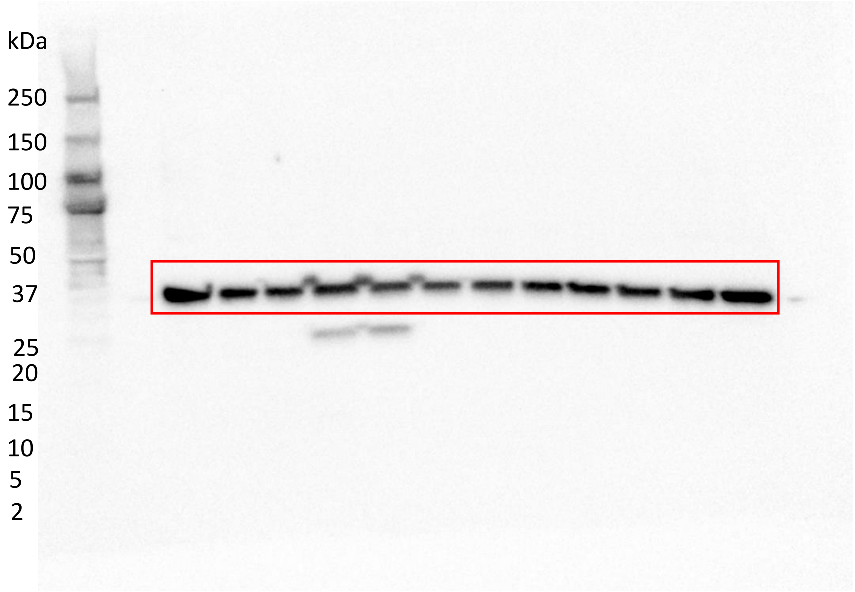

FIGURE 4: Amyloid beta (oligomeric; 50-75kDa)

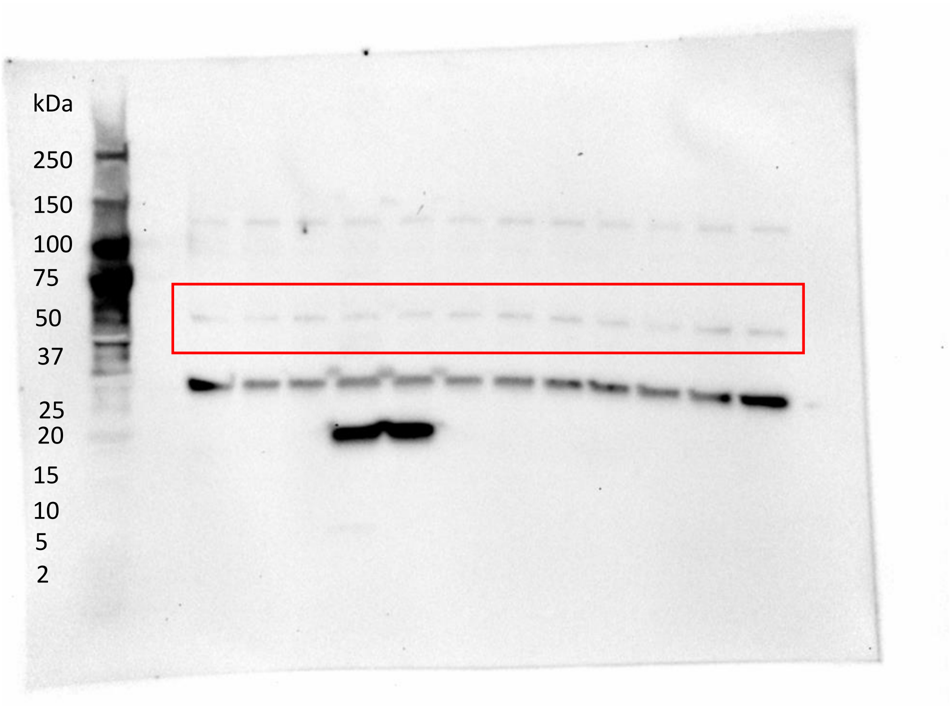

Figure 5A

Cathepsin B (25, 30 and 40kDa)

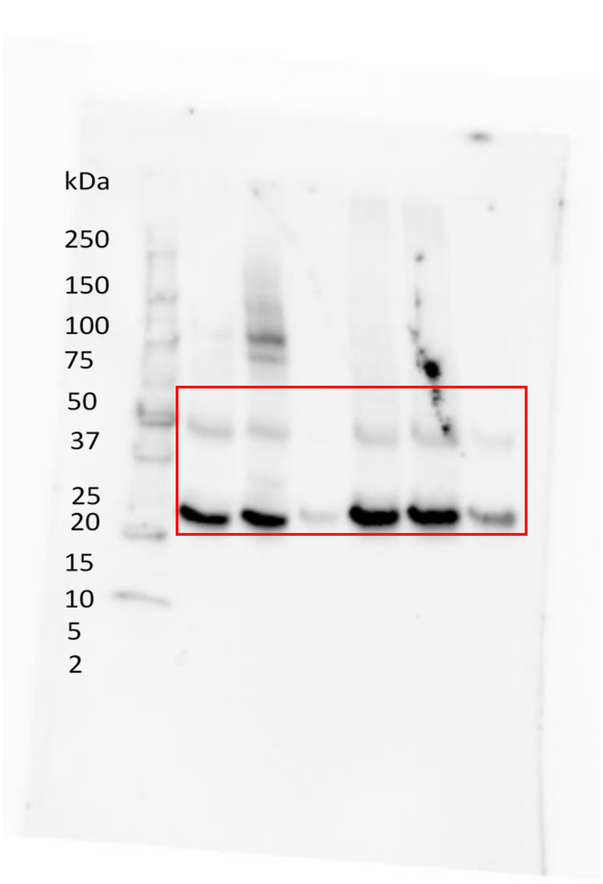

Serum amyloid P component (25kDa)

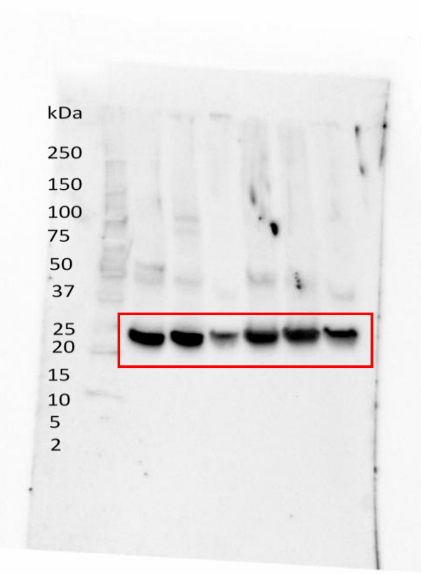

CD63 (53kDa)

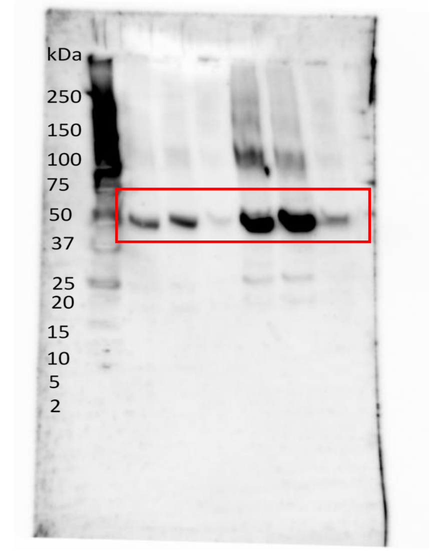

Hsp70 (53-70kDa)

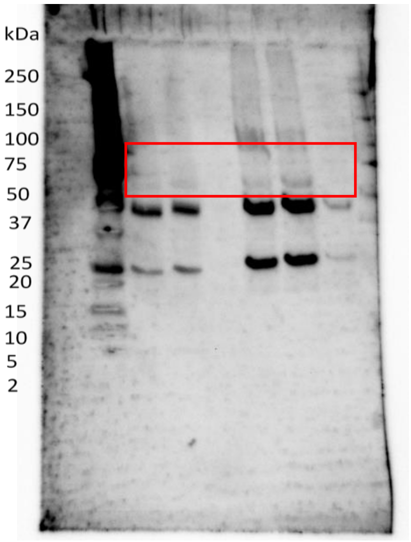

Figure 5F

Cathepsin B (25, 30 and 40kDa)

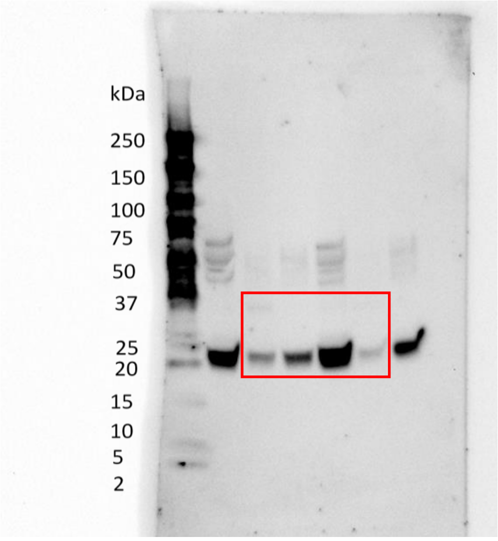

Serum Amyloid P Component (25kDa)

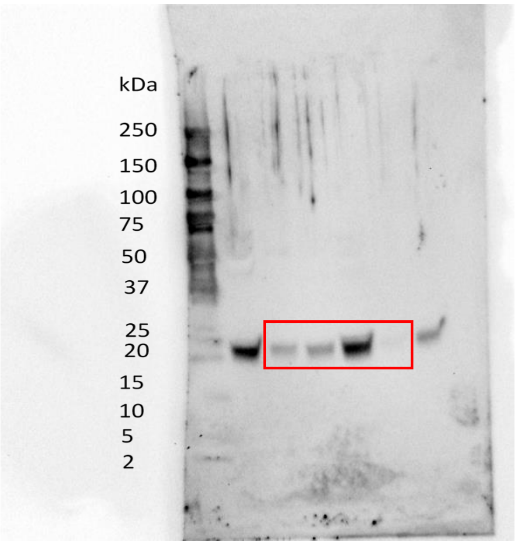

Hsp70 (53-70kDa)

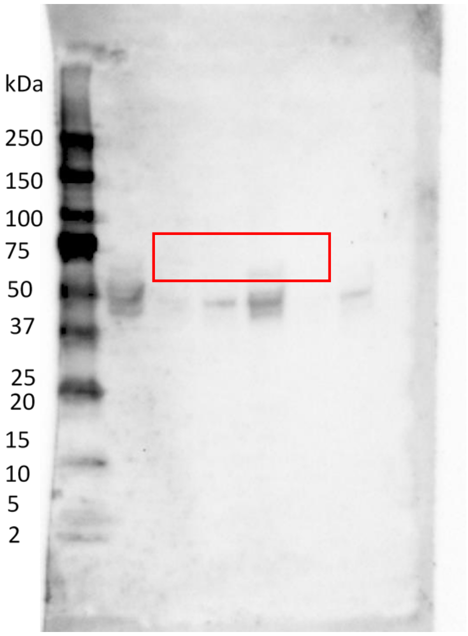

Supplement: Supplementary file 1 — HIV Infection Induces Extracellular Cathepsin B Uptake and Damage to Neurons supplementary information [file 41598_2019_44463_MOESM1_ESM.pdf]
